# miR-124-3p delivered by exosomes from heme oxygenase-1 modified bone marrow mesenchymal stem cells inhibits ferroptosis to attenuate ischemia–reperfusion injury in steatotic grafts

**DOI:** 10.1186/s12951-022-01407-8

**Published:** 2022-04-22

**Authors:** Longlong Wu, Xuan Tian, Huaiwen Zuo, Weiping Zheng, Xiang Li, Mengshu Yuan, Xiaorong Tian, Hongli Song

**Affiliations:** 1 School of Medicine, Nankai University, Tianjin, People’s Republic of China; 2 Tianjin First Central Hospital Clinic Institute, Tianjin Medical University, Tianjin, 300070 People’s Republic of China; 3 Department of Organ Transplantation, Tianjin First Central Hospital, School of Medicine, Nankai University, No. 24 Fukang Road, Nankai District, Tianjin, 300192 People’s Republic of China; 4 NHC Key Laboratory of Critical Care Medicine, Tianjin, 300192 People’s Republic of China; 5 Tianjin Key Laboratory of Organ Transplantation, Tianjin, People’s Republic of China

**Keywords:** Ferroptosis, Exosomes, Bone marrow mesenchymal stem cells, miR-124-3p, STEAP3, Heme oxygenase oxygen-1, Ischemia–reperfusion injury, Liver transplantation

## Abstract

**Background:**

Steatotic livers tolerate ischemia–reperfusion injury (IRI) poorly, increasing the risk of organ dysfunction. Ferroptosis is considered the initiating factor of organ IRI. Heme oxygenase oxygen-1 (HO-1)-modified bone marrow mesenchymal stem cells (BMMSCs) (HO-1/BMMSCs) can reduce hepatic IRI; however, the role of ferroptosis in IRI of steatotic grafts and the effect of HO-1/BMMSCs-derived exosomes (HM-exos) on ferroptosis remain unknown.

**Methods:**

A model of rat liver transplantation (LT) with a severe steatotic donor liver and a model of hypoxia and reoxygenation (H/R) of steatotic hepatocytes were established. Exosomes were obtained by differential centrifugation, and the differentially expressed genes (DEGs) in liver after HM-exo treatment were detected using RNA sequencing. The expression of ferroptosis markers was analyzed. microRNA (miRNA) sequencing was used to analyze the miRNA profiles in HM-exos.

**Results:**

We verified the effect of a candidate miRNA on ferroptosis of H/R treated hepatocytes, and observed the effect of exosomes knockout of the candidate miRNA on hepatocytes ferroptosis. In vitro, HM-exo treatment reduced the IRI in steatotic grafts, and enrichment analysis of DEGs suggested that HM-exos were involved in the regulation of the ferroptosis pathway. In vitro, inhibition of ferroptosis by HM-exos reduced hepatocyte injury. HM-exos contained more abundant miR-124-3p, which reduced ferroptosis of H/R-treated cells by inhibiting prostate six transmembrane epithelial antigen 3 (STEAP3), while overexpression of *Steap3* reversed the effect of mir-124-3p. In addition, HM-exos from cell knocked out for miR-124-3p showed a weakened inhibitory effect on ferroptosis. Similarly, HM-exo treatment increased the content of miR-124-3p in grafts, while decreasing the level of STEAP3 and reducing the degree of hepatic ferroptosis.

**Conclusion:**

Ferroptosis is involved in the IRI during LT with a severe steatotic donor liver. miR-124-3p in HM-exos downregulates *Steap3* expression to inhibit ferroptosis, thereby attenuating graft IRI, which might be a promising strategy to treat IRI in steatotic grafts.

**Graphical Abstract:**

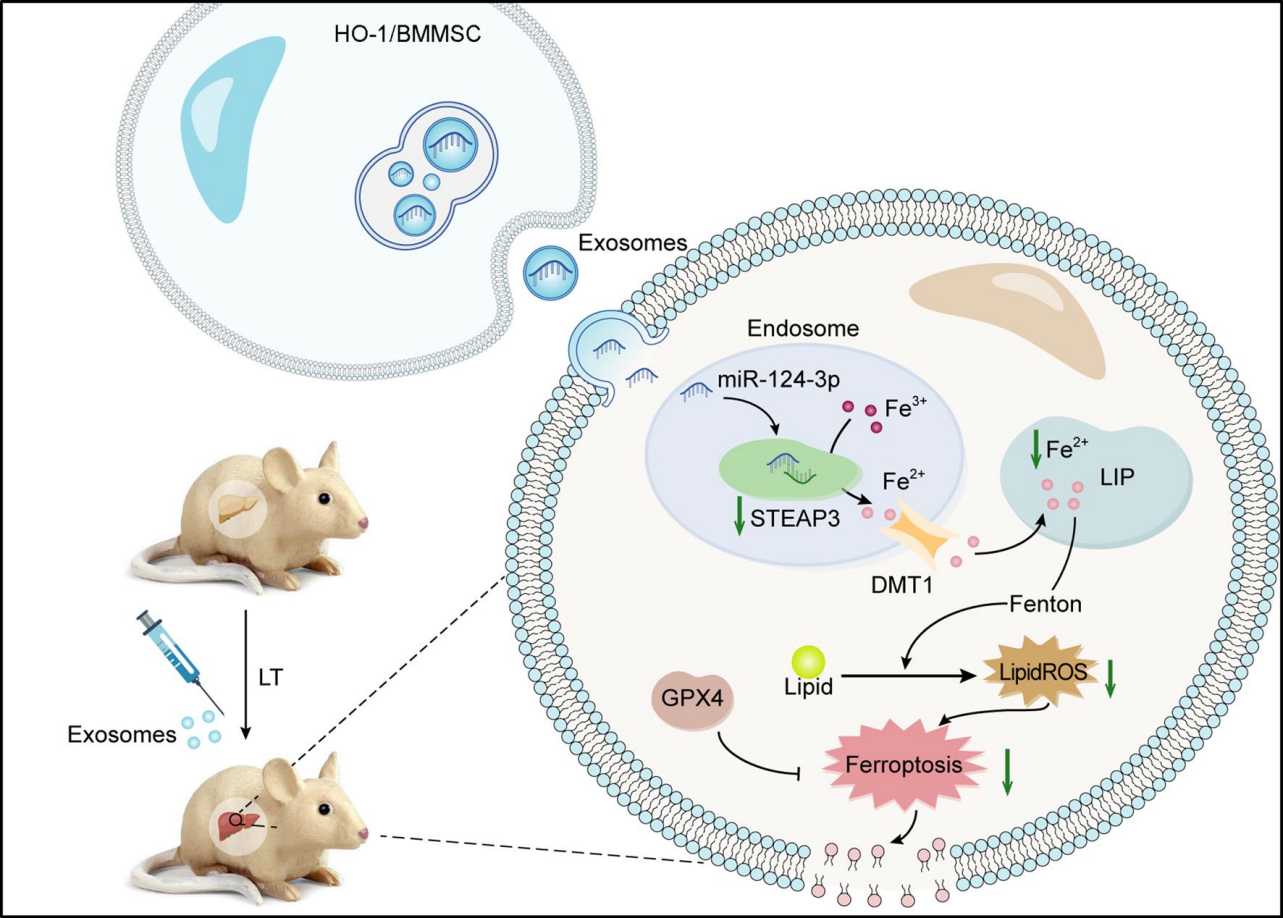

**Supplementary Information:**

The online version contains supplementary material available at 10.1186/s12951-022-01407-8.

## Introduction

Donor liver shortage is the main constraint to clinical liver transplantation (LT) at present. To alleviate this problem, the boundary of a marginal donor liver has gradually been expanded, and mildly steatotic livers have been used routinely in LT because of the high incidence of non-alcoholic fatty liver disease [1, 2]. A transplanted liver inevitably suffers from ischemia reperfusion injury (IRI), and severe hepatic steatosis aggravates liver IRI, which might lead to early graft dysfunction and primary dysfunction [3, 4]. Therefore, it is of great significance to clarify the mechanism of IRI in LT with a severe steatotic donor liver and to reduce graft IRI to expand the donor pool.

Recent studies have found that ferroptosis, a newly identified form of regulatory cell death, is an important driver of IRI in the liver and kidney [5, 6]. Ferroptosis refers to cell death dependent on iron and lipid peroxidation, including increased iron accumulation, an impaired lipid repair system, and lipid peroxidation, resulting in membrane destruction and cell death [5]. Iron chelating agents alleviated IRI in isolated mouse hearts by inhibiting ferroptosis [7], and the ferroptosis inhibitor ferrostatin-1 (Fer-1) alleviated IRI in the liver [6], suggesting that ferroptosis is a potential target to overcoming IRI in solid organ transplantation, including LT. Steatotic grafts face more severe IRI, while it is not known whether ferroptosis mediates steatotic graft IRI.

The immunomodulatory, repair, and pro-regeneration functions of mesenchymal stem cells (MSCs) mark them one of the potential treatment methods for IRI [8, 9]; however, their characteristics of heterogeneity and uncertain differentiation ability limit the clinical application of MSCs [10, 11]. Heme oxygenase oxygen-1 (HO-1) gene modification can improve the therapeutic effect of bone marrow mesenchymal stem cells (BMMSCs) [8, 12]; however, it is still difficult to completely solve all the problems associated with the clinical application of MSCs. The function of BMMSCs mainly depends on their paracrine function [13], while exosomes are important paracrine substances of BMMSCs. Exosomes mediate cell microcommunication by delivering mRNAs, non-coding RNAs, and proteins between cells [14, 15], and show low immunogenicity and are convenient for transplantation and storage. Therefore, exosome-based cell-free therapy has potential clinical application prospects [10, 16]. However, the biological composition and function of exosomes are largely influenced by the environment, and many studies have been devoted to the modification of exosomes by gene modification, biomaterial encapsulation, and electroporation to obtain more potent and stable cell-free drugs [17, 18]. Genetic modification is mainly targeted to exosome-producing cells and this may be one of the ways to enhance their exosome function. Our previous study found that the cargo profile of exosomes derived from HO-1-modified BMMSCs (HM-exos) changed, which significantly reduced the inflammatory injury of intestinal epithelial cells compared with the exosomes derived from unmodified BMMSCs (M-exos) [19], However, the potential role and mechanism of HO-1/BMMSC-derived exosomes on steatosis-donor liver IRI is unclear.

As an important component of exosomes, microRNAs (miRNAs) have been detected in exosomes from a variety of cell types [20], including MSCs. miRNAs mainly regulate biological process by inhibiting and degrading mRNA to regulate protein translation [21]. miRNAs in MSC-derived exosomes have been shown to play a role in several liver disease models, for example, adipose MSC-derived exosomes carrying miR-17 alleviated acute liver injury in mice by targeting thioredoxin-interacting protein and inhibiting the activation of inflammatory vesicles in hepatic macrophages [22]. This suggested that miRNAs are able to target multiple cells in the liver and regulate different protein types to influence disease progression. A study found that miRNAs mediate ferroptosis in hepatoma cells [23]. However, the role of miRNAs in exosomes in steatosis donor liver IRI and whether they regulate ferroptosis in steatosis hepatocytes are not yet known.

To date, few studies have directly used MSC-derived exosomes for liver transplantation treatment; however, it is clear that exosomes have advantages, such as better plasticity and lower risk compared with the cells themselves. In the present study, we used HO-1/BMMSC-derived exosomes to treat IRI in severe steatotic liver transplantation, and explored the role of ferroptosis in IRI in steatotic grafts, as well as the role and potential mechanism of regulation of ferroptosis by HO-1/BMMSC-derived exosomes. Our study provides a new therapeutic target for steatotic donor liver IRI and a new strategy for the application of exosomes in organ transplantation.

## Materials and methods

### Animals

The China Food and Drug Administration (Beijing, China) provided specific pathogen free (SPF) SD rats. The rats had free access to food and drink. All animals were treated humanely in accordance with the requirements of the National Institutes of Health Laboratory Animal Care and Use Guidelines (8th Edition). The animal experiments were approved by animal Ethics Committee of Nankai University (License No: 2021-SYDWLL-000331).

### Preparation and identification of HO-1/BMMSCs

BMMSCs were extracted and cultured according to our previous methods [9]. Cells were cultured in Dulbecco’s modified Eagle's medium (DMEM)/F12(Gibco at ThermoFisher Scientific, Waltham, MA, USA) containing 10% fetal bovine serum (FBS, Biowest, Nuaille, France) + 1% penicillin–streptomycin (Gibco). The third generation of BMMSCs was selected for *Hmox1* (encoding heme oxygenase 1 (HO-1)) gene transfection, using Ad/HO-1, which also carried the green fluorescent protein (GFP) (GeneChem, Shanghai, China). HO-1/BMMSCs were induced to undergo osteogenic and adipogenic differentiation. The HO-1/BMMSC phenotype was identified using flow cytometry, with anti-cluster of differentiation (CD)29, CD90, CD34, CD45, RT1A, and RT1B antibodies (BioLegend, San Diego, CA, USA). Expression of *Hmox1* was verified using quantitative real-time reverse transcription PCR (qRT-PCR) and western blotting.

### Exosome isolation and characterization

According to previous methods [24], exosomes were separated using differential centrifugation. BMMSCs or HO-1/BMMSCs were cultured in exosome-free FBS media (Sesh-biotech, Shanghai, China) for 72 h, and the supernatant was collected and centrifuged at 250×*g* for 15 min and 3000×*g* for 30 min, consecutively, to remove cell fragments. After 0.22 μm filtration and centrifugation at 110,000×*g* for 140 min at 4 ℃, the exosomes were deposited at the bottom of the ultracentrifuge tube. The exosomes were re-suspended in sterile phosphate-buffered saline (PBS), and the supernatant was retained after centrifugation at 10,000×*g* for 5 min. The obtained exosomes were temporarily stored at − 80 ℃. Western blotting was used to detect exosomes markers, and antibodies against CD9, tumor susceptibility 101 (TSG101), CD63, Calnexin (all ProteinTech, Wuhan, China), and CD81 (Abcam, Cambridge, UK) were used. The morphology of the exosomes was observed using transmission electron microscopy (TEM, Hitachi-HT7700, Tokyo, Japan). The particle concentration, size, and distribution were detected using nanoparticle tracking analysis (NTA, Malvern-NS300, Malvern, UK).

### Establishment of MCD model in rats

SD rats aged 7–8 weeks (230 ± 20 g) were selected and fed with a methionine and choline deficient (MCD) diet with free access to water. Liver tissue was collected 14 days later for hematoxylin and eosin (H&E) staining and oil red staining.

### Model of LT with severe steatotic donor liver and in vivo experimental design

The same doctor performed all the LTs in accordance with the formula developed by Kamada and Calne [25]. The steatotic livers were transplanted into normal SD rats (230 ± 20 g). The duration of the hepatic stage was (21 ± 1 min).

The animals were divided into seven groups: Sham group (Sham), PBS group, BMMSC group, HO-1/BMMSC group, M-exo group, HM-exo group, and Fer-1 group, with six rats in each group at each time point. In the Sham group, rats were subjected to abdominal opening and abdominal closing only. In the PBS group, 500 μL of PBS was injected via the portal vein after transplantation. In the BMMSC group, 2 × 10^6^ BMMSCs were injected through the portal vein after transplantation. In the HO-1/BMMSC group, 2 × 10^6^ HO-1/BMMSCs were injected through the portal vein. In the M-exo group, M-exos were injected via the portal vein (2.5 × 10^10^ particles in 500 μL of PBS). In the HM-exo group, HM-exos were also injected via the portal vein (2.5 × 10^10^ particles in 500 μL of PBS). The Fer-1 group was injected with Ferrostatin-1 (Fer-1) (10 mg/kg/d) via intraperitoneal injection after surgery (MCE, Shanghai, China). Rats in each group were sacrificed on postoperative day (POD) 1, POD3, and POD7, and liver tissue and serum were collected. An automatic biochemical analyzer was used to measure the serum levels of aspartate aminotransferase (AST), alanine aminotransferase (ALT), and total bilirubin (TBIL). The ultrastructure of the liver tissue was observed using transmission electron microscopy.

### In vivo biodistribution of exosomes

Exosomes were first labeled with 2 μL of chloromethyl (CM)-1,1'-dioctadecyl-3,3,3′3'-tetramethylindocarbocyanine perchlorate (DiI) dye (Invitrogen, Waltham, MA, USA). The labeled exosomes were incubated at 4 °C for 15 min in the dark, then cold PBS was added and the exosomes were centrifuged at 110,000×*g* for 70 min at 4 °C. After washing with PBS, the labeled exosomes were centrifuged again for 70 min. The exosomes (2.5 × 10^10^ particles in 500 μL of PBS) were injected into the rats. Liver tissue was taken for frozen sectioning on POD3 and nuclear stained using 4′,6-diamidino-2-phenylindole (DAPI) (Solarbio, Beijing, China). The distribution of the exosomes was observed under a fluorescence microscope.

### RNA sequencing (RNA-Seq)

The PBS group and the HM-exo liver tissue were selected for detection (n = 3). Total RNA of the sample was extracted, and identified and quantified using Nano Drop technology and an Agilent 2100 bioanalyzer (Agilent, Santa Clara, CA, USA). The mRNA library was then constructed and amplified with Phi29 to produce 100-base pair reads on the BGIseq500 platform (BGI, Shenzhen, China). SOAPnuke (V1.5.2) was used to filter the sequencing data, and Bowtie2 (V2.2.5) was used to compare the clean reads with the gene database established by Shenzhen Beijing Genomics Institute to calculate gene expression levels and identify differentially expressed genes (DEGs) (Fold change > 1.5; q-value < 0.05). The annotated DEGs were analyzed using Phyper (https://en.wikipedia.org/wiki/Hypergeometric_distribution) based on Gene ontology (GO) and Kyoto Encyclopedia of Genes and Genomes (KEGG) analysis. Gene set enrichment analysis (GSEA) was used to evaluate DEGs enriched for either negatively or positively correlated genes [26]. The RNA-Seq data were deposited in the NCBI Sequence Read Archive (PRJNA795289).

### Steatotic hepatocytes and hepatocytes hypoxia and reoxygenation (H/R) model

IAR20 cells were cultured in minimal essential medium (MEM) (Gibco) containing 10% FBS (Biowest); LO2 cells were cultured in 1640 medium (Gibco) containing 10% FBS. Cells were inoculated into 12-well plates and incubated with 20 μL of oleic acid and 5 μL of palm oil (Sigma-Aldrich, St. Louis, MO, USA) for 24 h to induce steatosis.

According to a previously published method [27], hepatocytes were temporarily deprived of oxygen by immersing them in mineral oil. In brief, IAR20 or LO2 cells were washed twice with PBS, soaked completely with mineral oil (Aladdin, Shanghai, China), and placed at 37 °C in a 5% CO_2_ incubator for 4 h. After repeated washing with PBS, MEM containing 10% FBS or 1640 medium containing 10% FBS were added for further culture for 6 h.

### Cell experiments

First, steatosis in IAR20 cells was induced and then the cells were treated with H/R or Erastin (20 μM) (MCE), Fer-1 (1 μM), and the apoptosis inhibitor Emricasan (30 μM) (MCE) for 24 h. In another experiment, both normal IAR20 cells and steatotic IAR20 cells were treated with H/R at the same time, and cells were collected 6 h later for detection. All experiments were repeated three times.

### Cellular uptake of exosomes

Exosomes were labeled with CM-DiI as described above, and then added to IAR20 and LO2 cells for 6 h. The cells were fixed using 4% paraformaldehyde for 30 min. The nuclei were stained with DAPI and then observed under a confocal microscope (FluoView™ -fv1000, Olympus, Tokyo, Japan).

### miRNA sequencing

Total RNA was extracted from M-exos and HM-exos using the Trizol reagent (Takara, Dalian, China), and RNA integrity was assessed using a 2100 bioanalyzer (Agilent Technologies). An Illumina TruSeq Small RNA kit (Illumina, San Diego, CA, USA) was used to construct the library, and a high-throughput sequencing platform was used to sequence the enriched 18–32 nt Small RNA fragments. The differentially expressed miRNAs in the two groups of exosomes were screened (Fold change > 1.5; *p*-value < 0.05), and miRDB [28] and miRWalk [29] databases were used to predict the target genes of the miRNAs, and KEGG enrichment analysis was performed for the identified target genes. The miRNA sequencing data were deposited in the Sequence Read Archive (PRJNA794949).

### Cell viability assay

According to the manufacturer's instructions, cell viability was measured using a Cell Counting Kit 8 (CCK-8) (Solarbio). In short, 5 × 10^3^ cells were inoculated into a 96-well dish, and 10 μL of CCK-8 solution was added and incubated at 37 ℃ for 3 h. The absorbance at 450 nm was then measured using a microplate meter.

### PI/FDA staining

The cells were digested with trypsin, the original supernatant was added to stop digestion, and the cells were collected by gently rubbing and shaking. After centrifugation, the cells were resuspended in 1 mL of PBS, and then transferred to a 12-well plate. Propidium iodide (PI) and fluorescein diacetate (FDA) dye (Sigma-Aldrich) were added (5 μL each), and incubated for 1 min in the dark, followed by observation under a fluorescence microscope.

### Lipid-ROS and ROS assay

Lipid-reactive oxygen species (ROS) levels were measured using BODIPY-C11 dye (Invitrogen). In brief, the cells were inoculated into a 12-well plate. On the second day, the culture medium was changed to fresh culture medium containing 5 µM BODIPY-C11, and incubated at 37 ℃ for 20 min, washed with PBS twice, and then used for analysis by flow cytometry. ROS detection was performed using a Reactive oxygen species assay kit (Beyotime Biotechnology, Shanghai, China). Cells in 12-well plates were digested with trypsin without EDTA (Solarbio), and then the cells were suspended in blank culture medium containing 10 μmol/L Dichloro-dihydro-fluorescein diacetate (DCFH-DA). The cells were incubated at 37 ℃ for 30 min, washed with PBS twice, and analyzed using flow cytometry.

### Malondialdehyde (MDA) assay

According to the manufacturer's instructions, a lipid peroxidation assay kit (Beyotime Biotechnology) was used to detect the MDA concentration in cells and liver tissues. A microplate reader was used to measure the absorbance at 532 nm.

### Iron assay

According to the manufacturer's instructions, intracellular and liver tissue Fe^2+^ levels were measured using an iron assay kit (Abcam). Samples were homogenized in an iron buffer, and the supernatant was retained and incubated with an iron reducing agent and an iron probe. The absorbance of the samples at 532 nm was measured using a microplate reader.

### Labile iron pool (LIP) assay

According to a previously published method [30], the LIP was detected using Calcein-acetoxymethyl (AM; Beyotime Biotechnology). The cells were incubated with 2 μM Calcein-AM at 37 °C for 8 min, the cells were then suspended in Hank’s equilibrium salt solution, the concentration was adjusted to 1 × 10^6^, and the samples were incubated with Trypan blue. The fluorescence intensity was measured at 488 nm for excitation and 517 nm for emission, and then 100 μM 2, 2 bipyridine (Sigma-Aldrich) was added and incubated for 10 min. The fluorescence changes in each sample were calculated after the addition of 2, 2 bipyridine.

### Transfection of miRNA mimics and inhibitors, siRNA and plasmids

miRNA mimics, inhibitors, and small interfering RNAs (siRNA) were designed and synthesized by Genepharma (Jiangsu, China). The detailed sequences are shown in Additional file 2: Table S1. Lipofectamine 3000 (Invitrogen) was used to transfect the cells. In brief, the cells were inoculated into 6-well plates, and when the degree of fusion was 60–70%, the cells were transfected with 10 μL of negative control (NC)-mimic and miR-124-3p-mimic; 5 μL of NC-inhibitor and miR-124-3p-inhibitor; and 5 μg of *Steap3*-siRNA (an siRNA targeting the mRNA encoding prostate six transmembrane epithelial antigen 3 (STEAP3), respectively. A plasmid expressing the *Steap3* gene was constructed (GeneChem) and 2 μg of this plasmid was transfected into IAR20 cells using Lipofectamine 3000 to overexpress *Steap3*.

### Dual-luciferase reporter assay

The 3' UTR sequence of *Steap3* was inserted into the pmirGLO vector as the wild-type (WT), and the predicted binding target of miR-124-3p in the *Steap3* 3'-UTR was mutated in the pmirGLO vector as the mutant (MT) (Genepharma). HEK-293T cells were co-transfected with luciferase reporter plasmid and NC-mimic or miR-124-3p-mimic. At 48 h after transfection, the firefly luciferase activity was determined and normalized by the Renilla luciferase activity.

### Immunohistochemistry and immunofluorescence staining

According to a previous published method [9], immunohistochemical staining was performed to detect glutathione peroxidase 4 (GPX4) (ProteinTech), 4-hydroxynonenal (4-HNE) (Abcam), STEAP3 (Abcam), and Myeloperoxidase (MPO) (ProteinTech) expression in liver tissue. Immunofluorescence staining of hepatocytes were also performed in accordance with previously published methods [11]. In brief, IAR20 or LO2 cells were inoculated into 12-well plates, treated according to the designated groups, and stained with primary and secondary antibodies according to the manufacturer’s instructions. DAPI was added to label the nuclei and the cells were observed under a fluorescence microscope.

### qRT-PCR

Total RNA was extracted from exosomes, cells, and tissues using the Trizol reagent (Takara). TB Green® Premix Ex Taq™ and PrimeScript™ RT reagent Kit with gDNA Eraser (Takara) were used for reverse transcription and amplification. The mRNA and miRNA levels were normalized to the expression of *Actb* (encoding β-actin) or U6 and calculated via the standard 2 − ΔΔCt method [31]. The sequences of the primers used in this study are shown in Additional file 2: Tables S2 and S3. All primers were synthesized by Sangon Biotech (Shanghai, China).

### Western blotting

Radioimmunoprecipitation assay lysis buffer (Beyotime Biotechnology) containing protease inhibitors was used to extract proteins from tissues and cells. Western blotting was performed according to our previous approach [9], using antibodies targeting HO-1, GPX4 (both ProteinTech), STEAP3 (Abcam), glyceraldehyde-3-phosphate dehydrogenase (GAPDH) (Cell Signaling Technology, Danvers, MA, USA), and divalent metal transporter 1 (DMT1) (Santa Cruz Biotechnology, Santa Cruz, CA, USA). Image J 7.0 software (NIH, Bethesda, MD, USA) was used to analyze the image grayscale values and calculate relative protein expression.

### Statistical analysis

The data were analyzed using GraphPad Prism 8.0 (GraphPad Software, Inc., La Jolla, CA, USA). All experiments were performed independently at least three times. Data are expressed as the mean ± standard deviation. The mean values of two groups were compared using an unpaired Student's *t*-test. Comparison among groups was performed using one-way analysis of variance (ANOVA). *P* < 0.05 was considered statistically significant.

## Results

### HM-exos alleviate IRI in LT with a severe steatotic donor liver

**Fig. 1:**
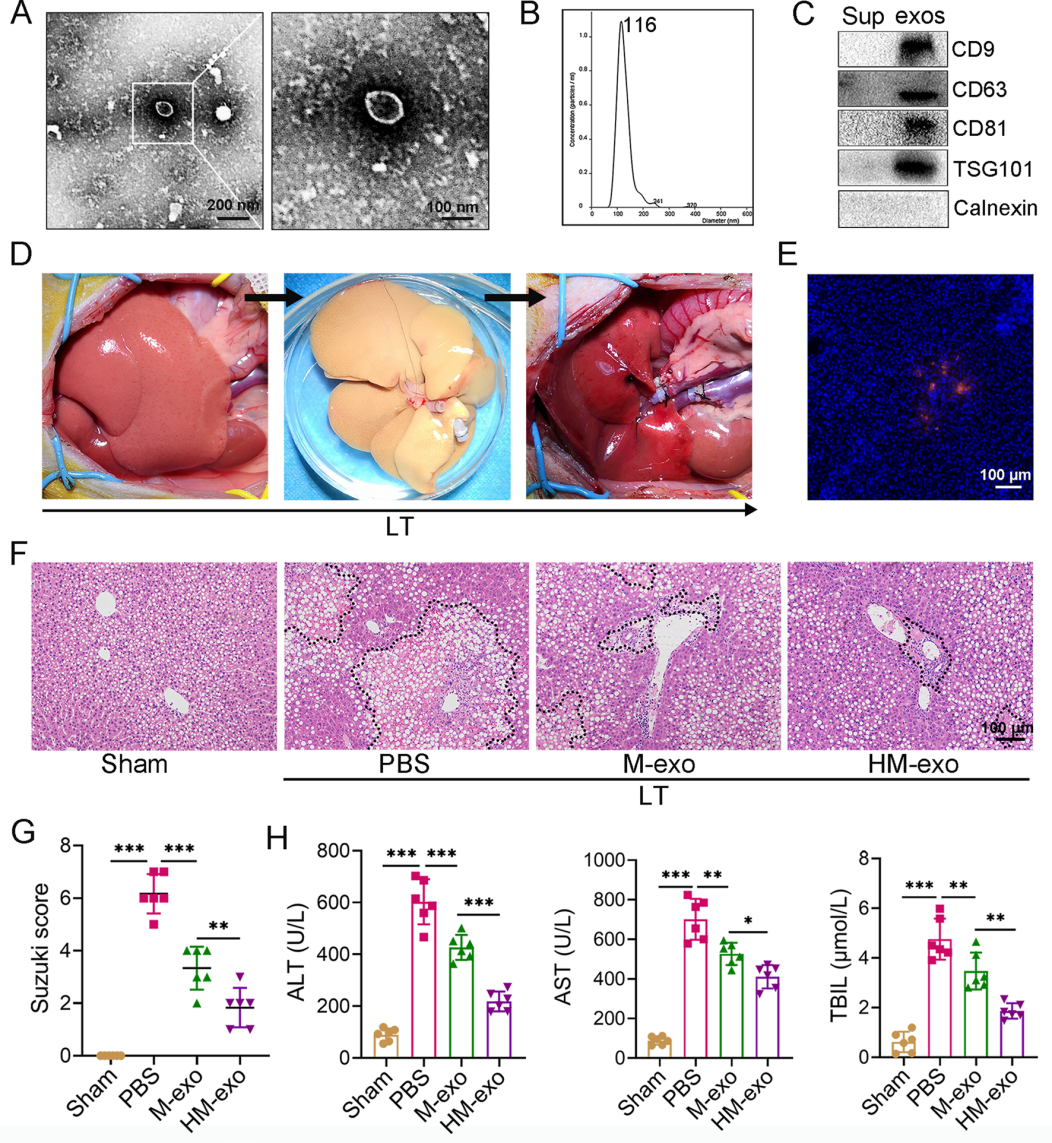
HM-exo alleviates IRI in LT with severe steatotic donor liver. **A** Morphology of exosomes under TEM: exosomes appear as typical bilayer membrane vesicles. **B** The particle size of exosomes, as detected using NTA (n = 3). **C** Western blotting detection of CD9, CD63, CD81, TSG101, and Calnexin levels in the supernatant (Sup) and exosomes (exos) (n = 3). **D** Complete procedure of liver transplantation with a severe steatotic donor liver. **E** Fluorescence images showing the biodistribution of CM-DiI (red) labeled exosomes in liver tissues. **F** Representative H&E staining images of liver tissues from different treatment groups (PBS, M-exo, and HM-exo), with the black dotted line representing necrotic areas. (n = 6). **G** Severity of the liver injury was assessed using histological sections of the liver according to the Suzuki liver injury scoring criteria (n = 6). **H** Levels of serum ALT, AST, and TBIL in each group (n = 6). *P < 0.05, **P < 0.01, ***P < 0.001. LT: Liver transplantation

BMMSCs transfected with HO-1 showed a long spindle shape, displayed green fluorescence (Additional file 1: Fig. S1A, B), expressed stem cell-specific biological markers (Additional file 1: Fig. S1C), and could induce osteogenic and adipogenic differentiation (Additional file 1: Fig. S1D, E). Meanwhile, HO-1/BMMSCs expressed high levels of HO-1 mRNA and protein (Additional file 1: Fig. S1F-H). The supernatants of BMMSCs and HO-1/BMMSCs were collected to obtain exosomes by differential centrifugation. TEM showed the exosomes as typical bilayer membrane vesicles (Fig. 1A). NTA analysis showed that the mean particle size of the exosomes was 118.3 ± 12.7 nm (Fig. 1B). The concentration of exosomes was 1.1 × 10^11^ particles per milliliter. Exosomes contained protein markers CD9, CD63, CD81, and TSG101, but did not contain Calnexin (Fig. 1C). A rat model of severe liver steatosis was established, and Oil red O staining showed 70–80% hepatocyte steatosis, which was considered as severe liver steatosis (Additional file 1: Fig. S2A). Using normal SD rats as recipients, we successfully performed LT with severe steatotic donor livers (Fig. 1D). Injection of CM-DiI dye-labeled exosomes caused the exosomes to be enriched in the rat liver (Fig. 1E).

We compared the liver damage at POD1, POD3, and POD7, and found extensive necrosis in the liver at POD3, and the Suzuki score showed the most severe tissue damage and significantly increased liver enzymology (Additional file 1: Fig. S2B–D). Therefore, POD3 was selected for subsequent experiments. We found that treatment with both BMMSCs and HO-1/BMMSCs reduced tissue necrosis on POD3 (Additional file 1: Fig. S2E), and the Suzuki score, and transaminase and bilirubin levels were reduced significantly (Additional file 1: Fig. S2F, G). Injection of HO-1/BMMSCs reduced the IRI significantly compared with injection of BMMSCs. In addition, treatment with M-exos and HM-exos also reduced the injury of grafts significantly on POD3, and treatment with HM-exos further reduced the necrotic area of the liver (Fig. 1F); the Suzuki score and the transaminase and bilirubin levels were significantly lower than those after M-exo treatment (Fig. 1G, H). These results indicated that the therapeutic effect of exosomes derived from stem cells was similar to that of stem cells themselves, and that HM-exos had a better therapeutic effect on the IRI of the grafts compared with M-exos.

### Inhibition of ferroptosis attenuates IRI in steatotic grafts

**Fig. 2:**
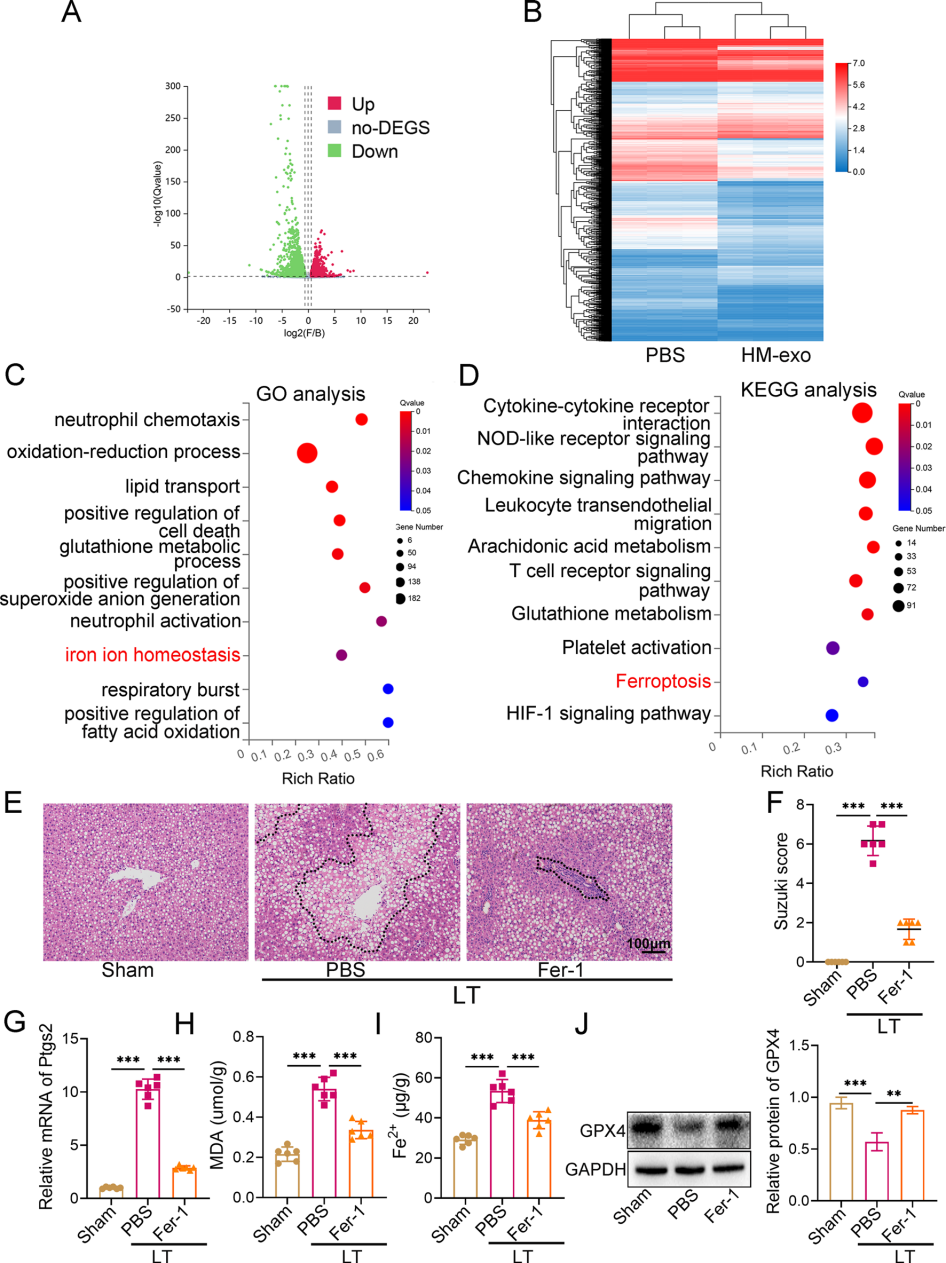
Ferroptosis mediates IRI in LT with a steatotic donor liver. **A** Volcano plot and heat map (**B**) showing the DEGs (Fold change > 1.5; q-value < 0.05;) in the PBS and HM-exo groups (n = 3). **C** GO analysis of DEGs was carried out, and 10 enriched biological processes are shown. **D** KEGG analysis of DEGs was carried out, and 10 of the pathways with significant enrichment are shown. **E** H&E staining images of liver tissue and the liver Suzuki score. **F** In the Sham group, PBS group, and Fer-1 group (n = 6). **G** Expression of liver *Ptgs2* mRNA in each group, with *Actb* (β-actin) as the internal reference (n = 6). **H** Levels of liver MDA and Fe^2+^ (**I**) in each group (n = 6). **J** The level of GPX4 in each group, with GAPDH as the internal reference (n = 6). *P < 0.05, **P < 0.01, ***P < 0.001. LT: Liver transplantation

To explore the possible mechanism by which exosomes alleviate IRI, liver tissues from the PBS group and the HM-exo group were selected for RNA-Seq analysis, and DEGs between the two groups were screened. Figure 2A shows the volcano map of the DEGs, and (Fig. 2B) shows the heat map of the relative expression levels of the DEGs. GO and KEGG analysis of the DEGs were further performed. Figure 2C and Additional file 2: Table S4 show 10 biological processes enriched in the GO analysis, including regulation of iron ion homeostasis, positive regulation of superoxide anion generation, and other processes. Figure 2D and Additional file 2: Table S5 show 10 signaling pathways, including ferroptosis, obtained by the KEGG analysis.

We verified the results of the enrichment analysis experimentally: The steatotic grafts showed extensive necrosis after transplantation and the Suzuki score increased (Fig. 2E, F). The levels of ferroptosis markers including *Ptgs2* (encoding prostaglandin-endoperoxide synthase 2), MDA, and Fe^2+^ increased significantly (Fig. 2G–I), while the levels of GPX4, which is involved in antioxidant activity, decreased significantly (Fig. 2J). Application of the ferroptosis inhibitor Fer-1 reduced the levels of the above ferroptosis markers significantly, increased the content of GPX4, and alleviated liver injury significantly (Fig. 2E–J). These results showed that inhibition of ferroptosis reduced the IRI of the grafts.

### Ferroptosis occurred in H/R treated steatotic hepatocytes

**Fig. 3:**
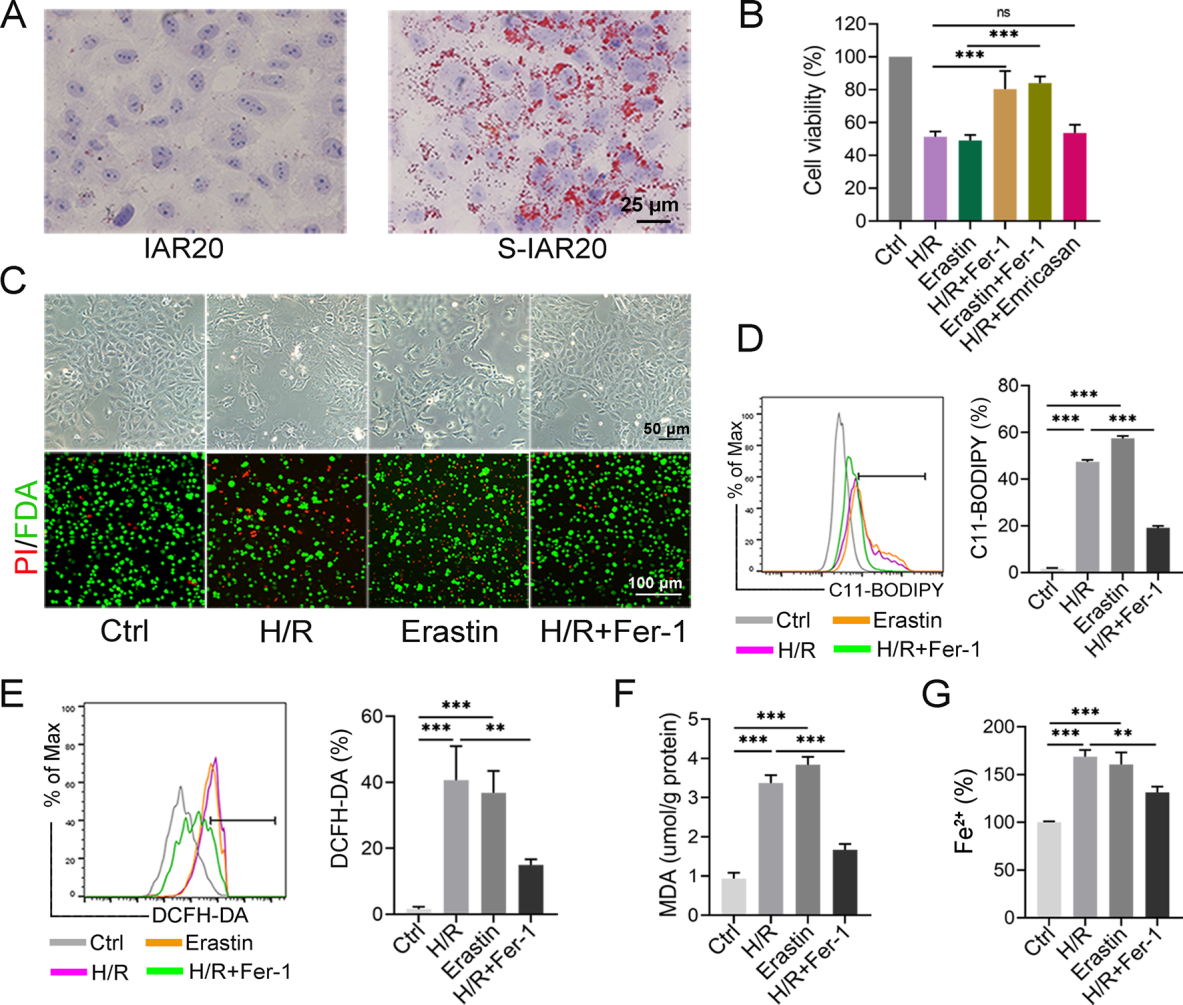
Ferroptosis occurred in H/R treated steatotic IAR20 cells. **A** Oil red O staining of normal IAR20 cells and steatotic IAR20 cells (S-IAR20). **B** Steatotic IAR20 cells treated with H/R or Erastin (20 μM), with or without Fer-1 (1 μM) or Emricasan (30 μM), respectively, and CCK-8 assays of cell viability. **C** The morphology of each group was observed under a phase contrast microscopy, and dead (red) and live (green) cells were identified by PI/FDA staining. **D** The levels of Lipid-ROS and ROS (**E**) were detected using C11-BODIPY and DCFH-DA, respectively. **F** The levels of MDA and Fe^2+^ (**G**) in each group of cells (n = 3). *P < 0.05, **P < 0.01, ***P < 0.001. ns: not significant

To simulate the in vivo environment, IAR20 cells were first induced to undergo steatosis (Fig. 3A), followed by H/R treatment. We found that treatment with both H/R and the ferroptosis inducer Erastin reduced cell viability significantly, Fer-1 restored cell viability, while the apoptosis inhibitor Emricasan could not improve cell viability (Fig. 3B). In addition, H/R resulted in cell swelling, rupture, and a large amount of cell death, while Fer-1 treatment significantly reduced cell death (Fig. 3C). We further found that, similar to Erastin, H/R treatment led to a significant increase in the levels of cellular Lipid-ROS, MDA, and Fe^2+^, while Fer-1 significantly reduced their levels, and could inhibit the significantly increased ROS levels after H/R treatment (Fig. 3D–G). The above results suggested that H/R leads to the occurrence of ferroptosis in steatotic cells, while inhibition of ferroptosis could attenuate the cell damage caused by H/R. In addition, Lipid-ROS, ROS, and MDA levels were significantly higher in steatotic IAR20 cells after H/R treatment compared with those in normal IAR20 cells (Additional file 1: Fig. S3A–C), and cell death was more frequent (Additional file 1: Fig. S3D).

### HM-exos attenuate the degree of ferroptosis in steatotic hepatocytes after H/R treatment

**Fig. 4:**
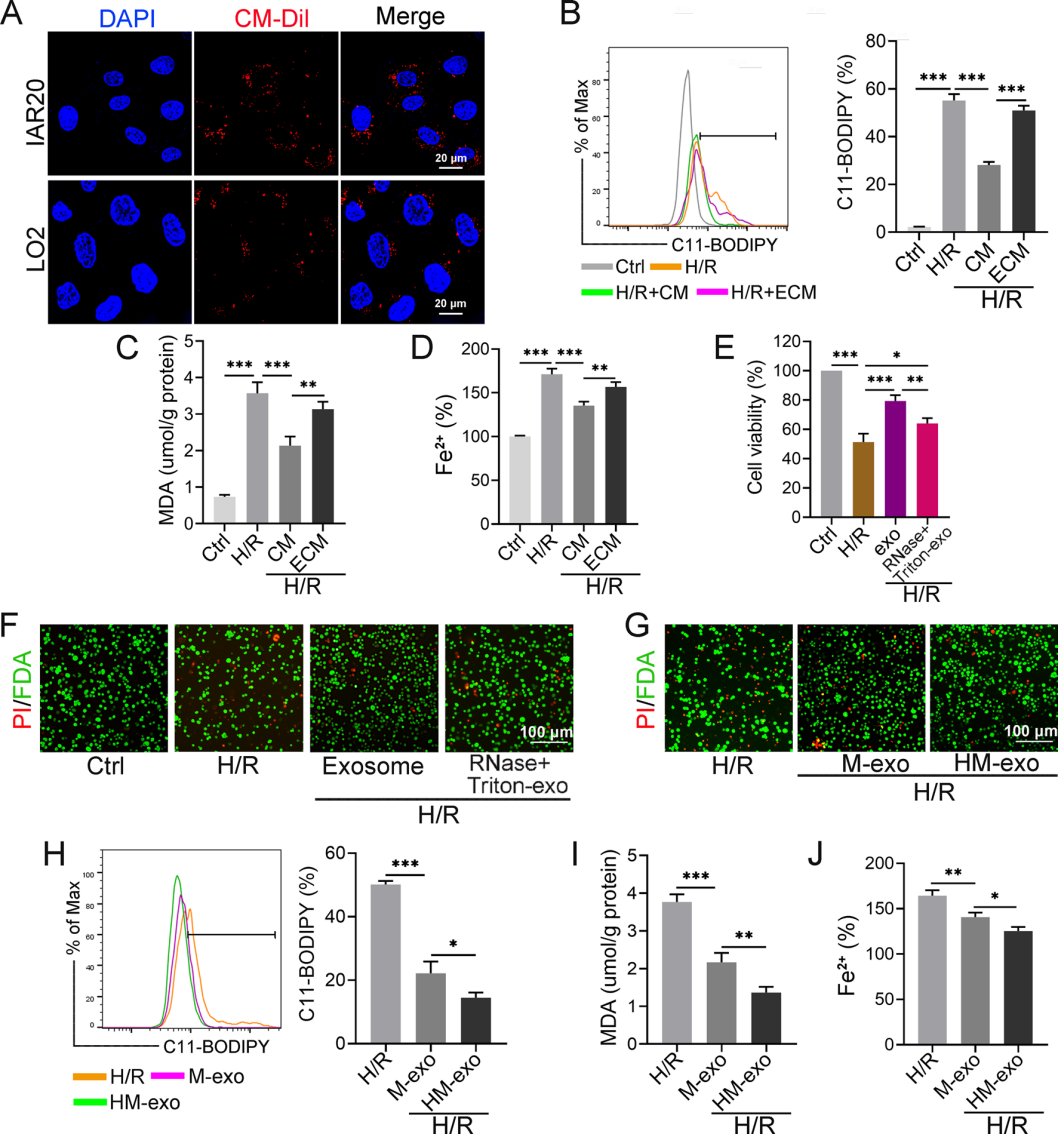
HM-exos attenuate ferroptosis in steatotic hepatocytes after H/R treatment. **A** Fluorescence images showing the uptake of CM-DiI-labeled (red) exos by IAR20 and LO2 cells, respectively. **B** The steatotic IAR20 cells treated with H/R were added to CM or ECM, respectively, and cultured for 6 h. The levels of Lipid-ROS were detected using C11-BODIPY. **C** The levels of MDA and Fe^2+^ (**D**) in cells after CM or ECM intervention. **E** The steatotic IAR20 cells treated with H/R were added to PBS, exosomes (1.5 × 10^9^ particles), and RNase + Triton-treated exosomes (1.5 × 10^9^ particles), respectively, and cultured for 6 h. The viability of each group of cells was detected using a CCK-8 assay. **F** PI/FDA staining identifying dead (red) and live (green) cells in each group. **G** H/R-treated steatotic IAR20 cells were added with PBS, M-exos (1.5 × 10^9^ particles), and HM-exos (1.5 × 10^9^ particles), respectively; exosomes were added during reoxygenation and cultured for 6 h. PI/FDA staining to identify dead (red) and live (green) cells. **H** The level of Lipid-ROS was detected after intervention with PBS, M-exos, and HM-exos. **I** The levels of MDA and Fe^2+^ (**J**) in cells after intervention with PBS, M-exos, and HM-exos (n = 3). *P < 0.05, **P < 0.01, ***P < 0.001. exo: exosomes

To study the protective effect of exosomes on H/R treated cells, the uptake of exosomes by two hepatocyte lines, IAR20 and LO2, was studied. Confocal microscopy images showed that CM-DiI labeled exosomes (red) were located in the cytoplasm (Fig. 4A), suggesting that IAR20 and LO2 cell could take up exosomes. We added conditioned medium (CM) and CM with the exosomes removed using differential centrifugation (ECM) to H/R treated IAR20 cells, respectively. CM treatment significantly inhibited the increase in Lipid-ROS, MDA, and Fe^2+^ levels after H/R, while treatment with ECM had no inhibitory effect (Fig. 4B–D), suggesting that the exosomes secreted by BMMSCs are the main substance that inhibits ferroptosis. In addition, we treated exosomes with RNase + Triton. Triton could destroy the bilayer membrane, and the released RNA was degraded by RNase. The protective effect of treated exosomes on cells was significantly weakened; however, cell viability was partially restored compared with that in the H/R group (Fig. 4E, F).

Next, we compared the effects of M-exos and HM-exos on cellular ferroptosis. The results showed that both of them could reduce cell death and the levels of Lipid-ROS, MDA, and Fe^2+^; however, HM-exos could further reduce the degree of cellular ferroptosis and decreased the rate of cell death compared with those after M-exo treatment (Fig. 4G–J), suggesting that HM-exos had a stronger inhibitory effect on ferroptosis.

### Characterizations of miRNA profiles in exosomes

**Fig. 5:**
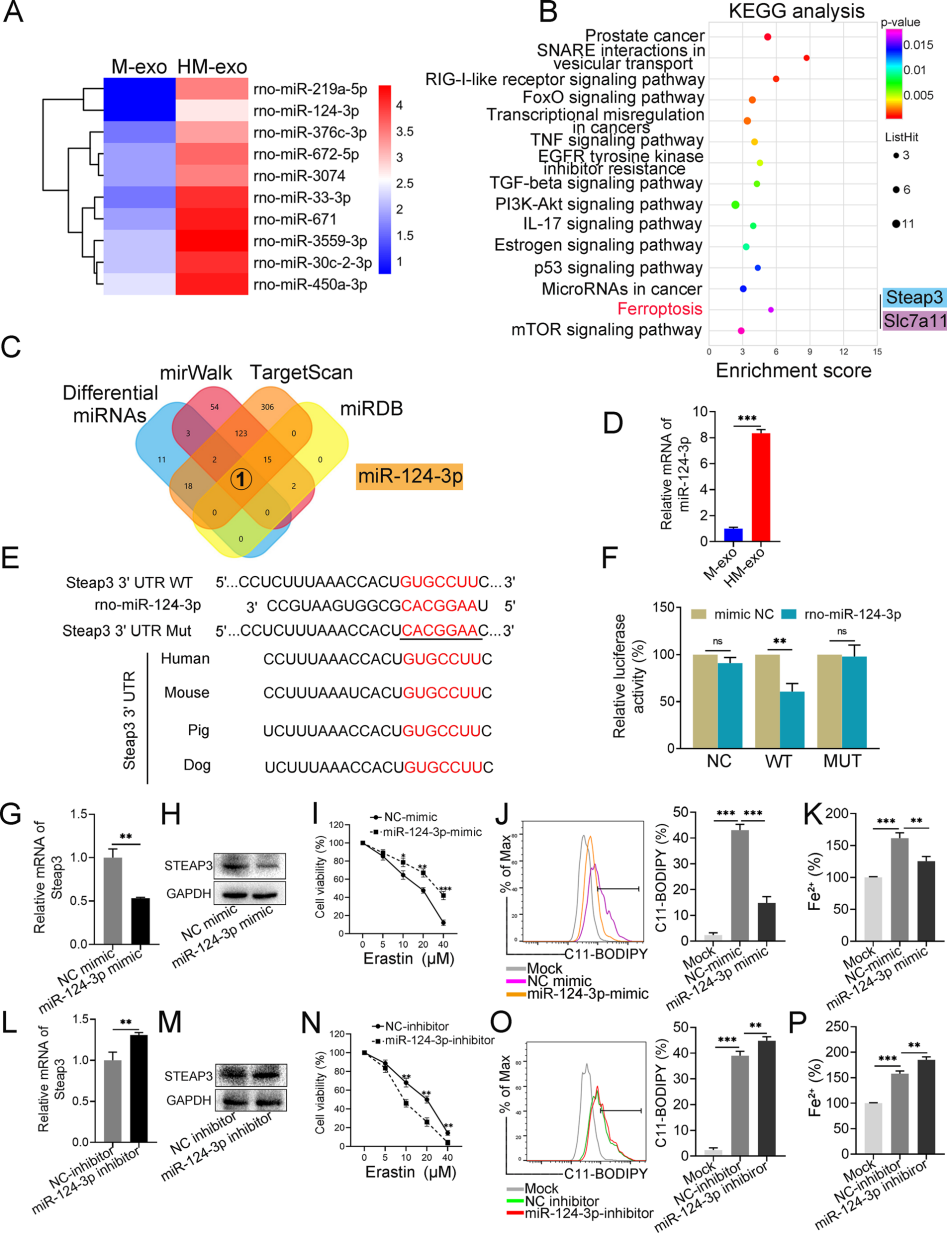
Characterization of miRNA profiles in exosomes and the role of miR-124-3p in ferroptosis. **A** Heatmap showing that 10 miRNAs were upregulated in the HM-exos group compared with the M-exos group (Fold change > 1.5; *p*-value < 0.05). **B** KEGG analysis of target genes of differential miRNAs, and 15 signaling pathways with significant enrichment are shown (*p*-value < 0.05). **C** Data in published databases were used to predict the microRNAs targeting *Steap3*, and those that further intersected with the miRNAs that were upregulated in the HM-exos. **D** Relative levels of miR-124-3p in M-exos and HM-exos. **E** Schematic sequence diagram of miR-124-3p targeting the wild-type or mutated 3' UTR of *Steap3*, the underlined sequence represents the 3'-UTR mutant seed sequence of *Steap3*, the binding site in *Steap3* is highly conserved in multiple species (bottom left). **F** Dual luciferase reporter gene assay verification of the targeted binding of miR-124-3p to the 3'-UTR of *Steap3* mRNA in HEK-293T cells. **G** Relative expression levels of STEAP3 mRNA and protein (**H**) in IAR20 cells overexpressing miR-124-3p. **I** Effect of Erastin on the viability of IAR20 Cells overexpressing miR-124-3p. **J** IAR20 cells were transfected with NC-mimic and miR-124-3p-mimic, respectively. The levels of Lipid-ROS and Fe^2+^ (**K**) were detected after H/R treatment. **L** Relative expression levels of STEAP3 mRNA and protein (**M**) in IAR20 cells transfected with the miR-124-3p-inhibitor. **N** Effect of Erastin on the viability of IAR20 cells transfected with the miR-124-3p inhibitor. **O** IAR20 cells were transfected with the NC-inhibitor and the miR-124-3p-inhibitor, respectively, and the levels of Lipid-ROS and Fe^2+^ (**P**) were detected after H/R treatment (n = 3). *P < 0.05, **P < 0.01, ***P < 0.001

miRNAs are important for exosomes to perform their biological functions; therefore, we performed an miRNA microarray analysis on M-exos and HM-exos, respectively. We focused on the upregulated miRNAs in HM-exos. Figure 5A shows the 10 miRNAs that were upregulated in HM-exos. KEGG analysis was performed on the target genes of the upregulated miRNAs, which revealed that genes encoding members of the ferroptosis pathway were enriched significantly (Fig. 5B; Additional file 2: Table S6). The enriched gene *Steap3* encodes prostate six transmembrane epithelial antigen 3 (STEAP3), which is involved in the regulation of ferroptosis by changing iron homeostasis [32]. We predicted the miRNAs that might target *Steap3* using related databases (Targetscan [33], mirWalk, and miRDB), and intersected the results with the data set of upregulated miRNAs in the HM-exo group, which identified miR-124-3p as a candidate miRNA (Fig. 5C). qRT-PCR confirmed that the miR-124-3p level in HM-exos was significantly higher than that in M-exos (Fig. 5D).

### miR-124-3p directly targets and negatively regulates *Steap3* to inhibit ferroptosis

Gene prediction tools suggested the presence of a binding site for miR-124-3p in *Steap3* that is highly conserved in many species (Fig. 5E). Dual luciferase reporter assays showed that miR-124-3p overexpression significantly inhibited the luciferase reporter gene activity of *Steap3* 3'-UTR WT compared with miRNA-NC, but did not affect the activity of the *Steap3* 3'-UTR mutant luciferase reporter gene, indicating that *Steap3* is a target of miR-124-3p (Fig. 5F). We further verified the effect of miR-124-3p on ferroptosis. Compared with the interference control, overexpression of miR-124-3p reduced the levels of STEAP3 mRNA and protein in IAR20 cells significantly (Fig. 5G, H) and increased the tolerance of the cells to Erastin (Fig. 5I). The elevated levels of Lipid-ROS and Fe^2+^ after H/R treatment were significantly suppressed after miR-124-3p overexpression (Fig. 5J, K). Inhibition of miR-124-3p resulted in a significant increase in STEAP3 mRNA and protein levels (Fig. 5L, M), and weakened the tolerance of the cells to Erastin (Fig. 5N). Ferroptosis was further aggravated after H/R treatment under conditions of miR-124-3p inhibition (Fig. 5O, P). In another hepatocyte line, LO2, overexpression of miR-124-3p similarly reduced STEAP3 mRNA and protein levels (Additional file 1: Fig. S4A, B), and the Fe^2+^ levels and lipid peroxidation responses were significantly inhibited (Additional file 1: Fig. S4C, D), suggesting that miR-124-3p is a negative regulator of ferroptosis.

### STEAP3 acts as a promoter of ferroptosis by regulating iron ion homeostasis

**Fig. 6:**
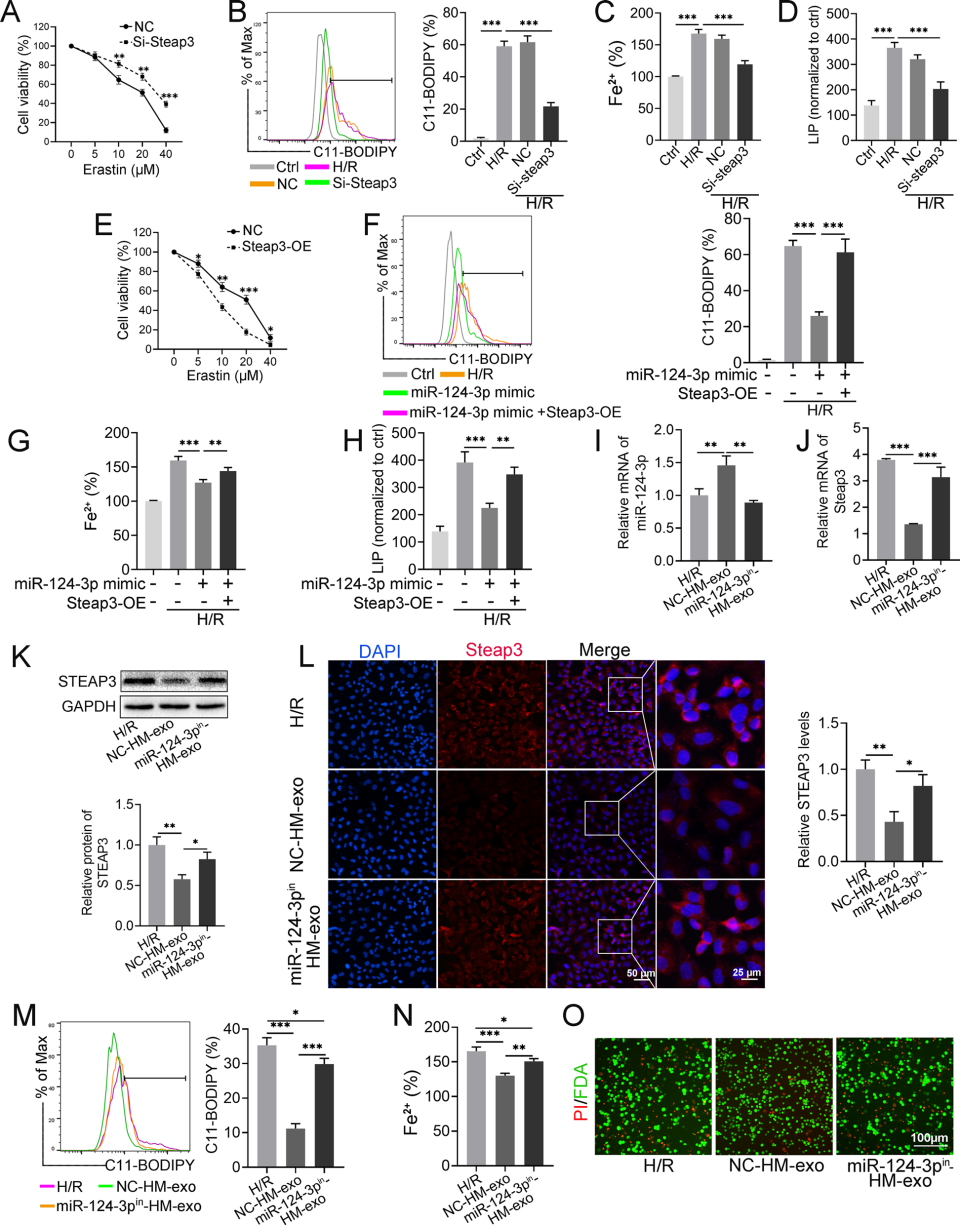
HM-exo delivery of miR-124-3p attenuates ferroptosis in H/R-treated cells. **A** Effect of Erastin on the viability of *Steap3*-silenced IAR20 cells. **B** The levels of Lipid-ROS and Fe^2+^ (**C**) in *Steap3*-silenced IAR20 cells after H/R treatment. **D** Calcein-AM method to determine the LIP levels in each group of cells. **E** Effect of Erastin on the viability of IAR20 cells overexpressing *Steap3*. **F** miR-124-3p mimic and *Steap*3-OE were transfected into IAR20 cells alone or simultaneously, and the cells’ Lipid-ROS, Fe^2+^ (**G**) and LIP (**H**) levels were measured after H/R treatment. **I** NC-HM-exos and miR-124-3p^in^-HM-exos intervention in IAR-20 cells, respectively, followed by treatment with H/R; exosomes were added during reoxygenation and cultured for 6 h, to detect the relative expression of miR-124-3p. **J** STEAP3 mRNA and protein (**K**) levels in each group. **L** Immunofluorescence showing STEAP3 levels in each group. **M** Lipid-ROS and Fe^2+^ (**N**) levels in each group. **O** PI/FDA staining to identify dead (red) and live (green) cells in each group (n = 3). *P < 0.05, **P < 0.01, ***P < 0.001. *Steap3*-OE: Plasmid vector overexpressing *Steap3*

As the target gene of miR-124-3p, we further explored the role of *Steap3* in ferroptosis. Firstly, siRNA was used to silence the expression of *Steap3* in IAR20 cells (Additional file 1: Fig. S5A, B). Compared with that in the NC group, silencing *Steap3* reversed the decrease in cell activity caused by Erastin (Fig. 6A). At the same time, the increased levels of Lipid-ROS and Fe^2+^ after H/R treatment were also significantly reduced (Fig. 6B, C). We also examined the level of intracellular LIP. H/R treatment increased the level of LIP, while silencing *Steap3* significantly reduced the level of LIP (Fig. 6D).

By contrast, we found that overexpression of *Steap3* (Additional file 1: Fig. S5C, D) increased the sensitivity of the cells to Erastin (Fig. 6E). In addition, transfection with miR-124-3p mimics significantly inhibited the increase in Lipid-ROS, Fe^2+^, and LIP caused by H/R; however, this inhibition was rescued by cotransfection with the *Steap3* overexpression vector (Fig. 6F–H). Furthermore, overexpression of *Steap3* resulted in a significant increase in Fe^2+^, LIP, and MDA, while the iron scavenger Deferoxamine mesylate (DFO) significantly reduced the levels of Fe2^+^, LIP, and MDA (Additional file 1: Fig. S6A–C) and decreased the rate of cell death (Additional file 1: Fig. S6D), indicating that STEAP3 is involved in the regulation of intracellular iron homeostasis.

### Delivery of miR-124-3p by HM-exos attenuates ferroptosis in H/R-treated cells

To explore the mechanism by which exosomes regulate ferroptosis, the miR-124-3p inhibitor was transfected into HO-1/BMMSCs to obtain HM-exos with low levels of miR-124-3p (miR-124-3p^in^-exo) (Additional file 1: Fig. S5E). NC-HM-exos or miR-124-3p^in^-exos were applied to H/R-treated IAR20 cells. The results showed that the content of miR-124-3p in cells increased significantly after NC-HM-exos treatment, while there was no significant change in the miR-124-3p^in^-exo group (Fig. 6I). In addition, NC-HM-exos significantly reduced the elevated STEAP3 mRNA and protein levels after H/R treatment, whereas the miR-124-3p^in^-exos reversed this effect (Fig. 6J–L).

We further investigated the effect of miR-124-3p^in^-exos on the ferroptosis of IAR20 cells. The miR-124-3p^in^-exos failed to significantly inhibit the elevated Lipid-ROS and Fe^2+^ levels after H/R treatment, and more cells died; however, the degree of ferroptosis was still relatively attenuated compared with that in the H/R group (Fig. 6M–O). Furthermore, based on the conservation of miR-124-3p and *Steap3* binding sites, we verified in LO2 cells that miR-124-3p^in^-exos failed to inhibit the upregulation of cellular *Steap3* after H/R treatment (Additional file 1: Fig. S7A), as demonstrated by the strong lipid peroxidation response (Additional file 1: Fig. S7B), the elevated MDA and Fe^2+^ levels (Additional file 1: Fig. S7C, D), and the increased cell death (Additional file 1: Fig. S7E). The above results suggested that HM-exos attenuate cellular ferroptosis by downregulating the level of *Steap3* through the delivery of miR-124-3p.

### HM-exos inhibit ferroptosis to attenuate neutrophil infiltration and the inflammatory response after transplantation

**Fig. 7:**
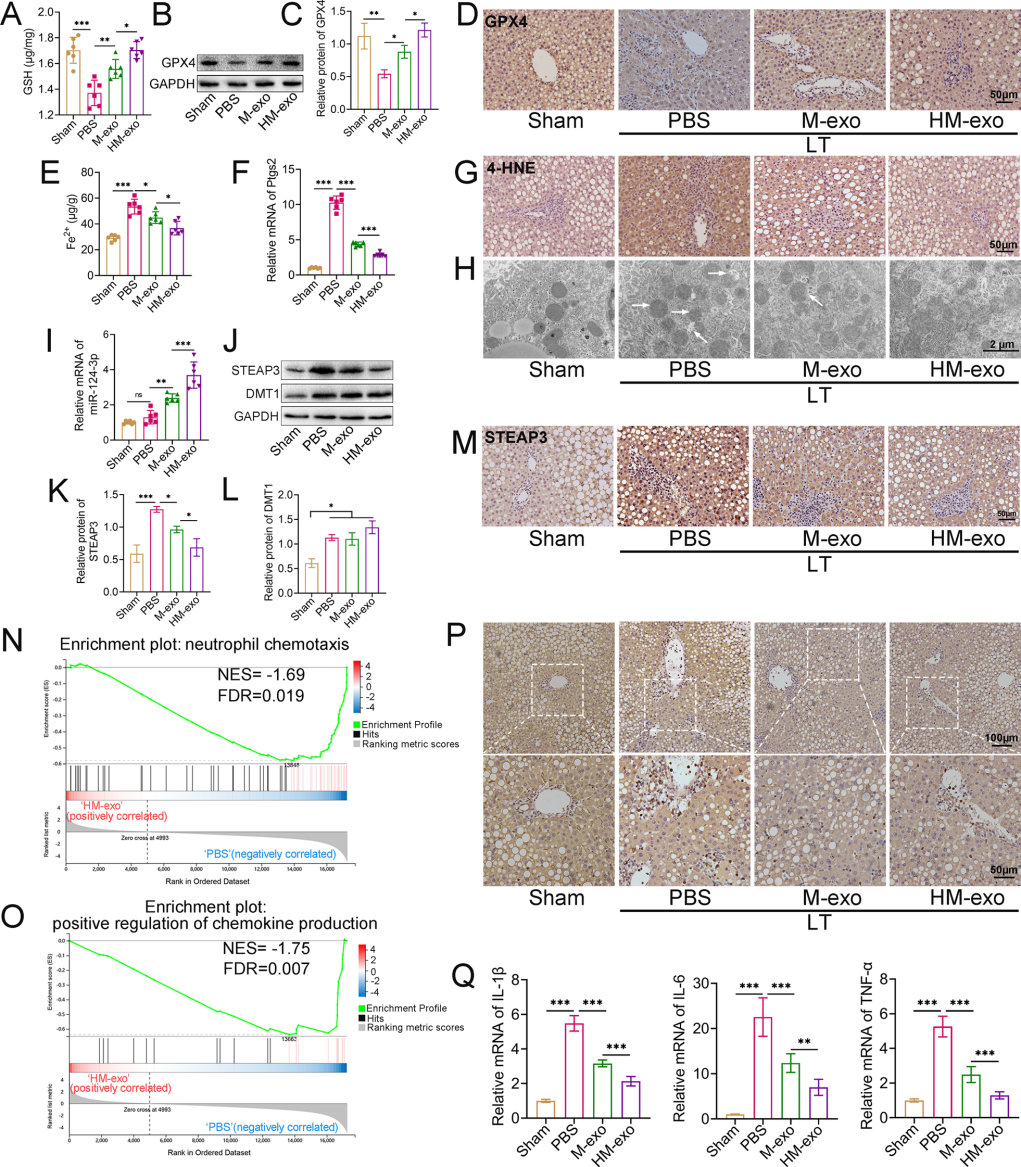
HM-exo-targeted inhibition of STEAP3 attenuates ferroptosis in steatotic grafts. **A** Rats were treated with PBS, M-exos, and HM-exos after LT, and the GSH levels of the liver tissues in each group were measured on POD3. **B**, **C** Protein levels of GPX4 in each group. **D** Immunohistochemistry showing the level of GPX4 in each group. **E** Fe^2+^ and relative expression of *Ptgs2* mRNA (**F**) in each group. **G** Immunohistochemistry showing the level of 4-HNE in each group. **H** The ultrastructure of the mitochondria in rat hepatocytes was observed using transmission electron microscopy, and the white arrows indicate the damaged mitochondria. **I** Relative expression of miR-124-3p in the liver tissues of each group, with U6 as the internal reference. **J**–**L** Protein levels of STEAP3 and DMT1 in liver tissues of each group. **M** Immunohistochemistry showing the level of STEAP3 in each group. **N**, **O** GSEA analysis of DEGs in the PBS and HM-exo groups. **P** MPO staining showing the infiltrated neutrophils in liver tissue of each group. **Q** Relative expression of *Il1*, *Il6*, and *Tnfa* mRNA in liver tissue of each group (n = 6). *P < 0.05, **P < 0.01, ***P < 0.001. LT: Liver transplantation

We further explored the effect of exosomes on ferroptosis in steatotic grafts. The results showed that the levels of glutathione (GSH) and GPX4 were decreased significantly in the PBS group (Fig. 7A–D), while the levels of Fe^2+^, *Ptgs*2 mRNA (Fig. 7E, F), and the levels of the lipid peroxidation product 4-HNE were significantly increased (Fig. 7G). Treatment with both M-exos and HM-exos attenuated the degree of ferroptosis in the liver; however, HM-exos further enhanced the antioxidant capacity of the liver, significantly reduced the levels of ferroptosis markers, and essentially normalized mitochondrial morphology (Fig. 7A–H). In addition, the application of HM-exos significantly increased the content of miR-124-3p in the liver (Fig. 7I), but significantly inhibited the elevated STEAP3 levels after transplantation (Fig. 7J–M). Furthermore, HM-exos failed to inhibit the upregulation of DMT1 (Fig. 7J, L). IRI triggers cell death to induce a sterile inflammatory response in tissues, and GSEA analysis of DEGs in the PBS and HM-exo groups showed that genes involved in neutrophil activation and chemokine signaling pathways were significantly downregulated (|normalized enrichment score (NES) |> 1, false discovery rate (FDR) < 0.25) in the HM-exo group (Fig. 7N, O). MPO staining confirmed that treatment with HM-exos reduced neutrophil granulocyte infiltration (Fig. 7P), and significantly decreased the elevated levels of *Il1* (encoding interleukin 1 (IL-1)), *Il6* (encoding interleukin 6 (IL-6)), and *Tnfa* (encoding tumor necrosis factor alpha (TNFα)) mRNA after transplantation (Fig. 7Q).

## Discussion

Steatotic livers are poorly tolerant to IRI and increase the risk of dysfunction in transplanted organs, which leads to the abandonment of many steatotic livers, thereby exacerbating the shortage of donors. Therefore, reducing the IRI in steatotic grafts is of great significance to expand their clinical application. In this study, we found that exosomes derived from HO-1/BMMSCs carry higher levels of miR-124-3p, which inhibits hepatocyte ferroptosis by targeting *Steap3* and reduces the IRI in LT with a severe steatotic donor liver.

Studies, including ours, have confirmed that BMMSCs attenuate hepatic IRI [12, 34], and HO-1-modified BMMSCs with significantly improved immunomodulatory and anti-stress capacities showed better therapeutic effects [8]. We found that HO-1/BMMSCs attenuated IRI in grafts significantly. However, the uncertainty of differentiation and risk of embolization present in cell therapy should be considered [10]. Recent studies have suggested that MSC-derived exosomes alleviate organ IRI through multiple mechanisms [24, 35]. Exosomes are the main method by which MSCs exert their biological functions and can avoid the potential problems caused by cell therapy [13]. In addition, modification of MSCs is one way to enhance the therapeutic effect of MSC-derived exosomes. Zheng et al. [36] found that pretreatment with the HO-1 inducer hemin could enhance the content of miR-183-5p in MSC-derived exosomes and enhance the inhibitory effect of exosomes on cardiomyocyte senescence. In this study, HM-exos reduced the IRI in grafts more significantly than M-exos, which confirmed that the modulation of HO-1 levels improved the therapeutic effect of cell-derived exosomes.

We analyzed enriched genes whose expression was altered after HM-exos treatment and found that the ferroptosis pathway was enriched significantly. Ferroptosis refers to iron-dependent lipid peroxidation, and the development of ferroptosis begins with iron accumulation, overproduction of ROS and lipid peroxidation, along with depletion of GSH and GPX4, which play an antioxidant effect in cells, ultimately leading to the development of ferroptosis [5, 37]. There is increasing evidence that ferroptosis is a major cause of organ IRI, Yamada et al. [6] found that post-LT iron overload is an independent risk factor for hepatic IRI and that ferroptosis mediates hepatic IRI and secondary inflammatory responses. In the present study, Fer-1 attenuated IRI in grafts significantly, suggesting that ferroptosis mediates IRI in grafts, and HM-exo-attenuated IRI might be related to its inhibition of ferroptosis.

Exosomes contain a variety of biomolecules, among which the relatively abundant miRNAs have been confirmed to be involved in the regulation of ferroptosis. Ding et al. [38] identified I/R-induced upregulation of miR-182-5p and miR-378A-3p, which led to the activation of ferroptosis in kidney injury by downregulating GPX4 and SLC7A11 levels. In this experiment, we found that the protective effect of exosomes on cells was significantly diminished after degradation of non-coding RNAs, indicating that non-coding RNAs, such as miRNAs, in exosomes might play a key role. In addition, HM-exos showed a more significant inhibition of ferroptosis than M-exos, which might have been caused by the increase in HO-1, which alters the components of BMMSC-derived exosomes, because HO-1 is known to regulate the expression of multiple miRNAs and miRNA processing enzymes [39].

We analyzed the miRNA profiles of M-exos and HM-exos, and identified a large number of differentially abundant miRNAs, and some target genes of these differential miRNAs were enriched in the ferroptosis pathway; therefore, the differentially abundant miRNAs in HM-exos might be the basis for their more significant ferroptosis inhibitory effects. Among the enriched genes, we identified *Steap3* as a potential target gene. As an iron reductase, STEAP3 can reduce Fe^3+^ in endosomes to Fe^2+^, and participates in mediating cellular iron homeostasis [40, 41]. The disturbance of cellular iron metabolism is a direct trigger of ferroptosis. Excessive Fe^2+^ induces extensive lipid peroxidation through the Fenton reaction, forming agent hydroxyl radicals, which results in the occurrence of ferroptosis. However, iron chelators (such as deferoxamine) inhibit ferroptosis by scavenging iron in cells [5, 42]. However, in tumor therapy, Wang et al. [43] used the generated hydroxyl radicals to enhance the chemodynamic treatment of tumors. Elevated STEAP3 leads to oxidative damage of stored erythrocytes through lipid peroxidation [44]. Liu et al. [32] demonstrated that silencing of *Steap3* blocked Erastin or RSL3 (a ferroptosis activator)-induced cell death. In vivo, both iron overload and acidosis occurring during reperfusion favor the reduction of excess iron to Fe^2+^ by STEAP3, and further enhance the Fenton reaction. In this study, silencing of *Steap3* attenuated ferroptosis in H/R-treated cells significantly, while overexpression of *Steap3* had the opposite effect. In addition, unused Fe^2+^ is stored temporarily in the cytoplasmic LIP, which responds to cellular free iron levels, and the expansion of LIP makes cells more sensitive to ferroptosis [30]. We found that overexpression of *Steap3* significantly increased the level of LIP, i.e., upregulation of STEAP3 led to a significant increase in reduction-generated Fe^2+^, and the excess Fe^2+^ led to LIP expansion, which further exacerbated the Fenton reaction, eventually leading to cell ferroptosis.

Based on published database prediction, we found that differentially expressed miR-124-3p may be a potential candidate miRNA targeting *Steap3*. miRNA activity leads to mRNA degradation and/or translation inhibition by targeting the 3' UTR of mRNA. We confirmed that the miR-124-3p mimic reduced the expression of *Steap3* significantly, and luciferase reporter assays further corroborated their targeting relationship. Li et al. [45] found that BMMSC-derived exosome miR-124-3p ameliorated spinal cord ischemia–reperfusion injury-induced neurological damage by inhibiting *Ern1* (encoding endoplasmic reticulum to nucleus signaling 1) and promoting M2 macrophage polarization. Thus, the higher abundance of miR-124-3p in HM-exos might underlie their more significant protective effects. As we hypothesized, the miR-124-3p mimic attenuated cellular ferroptosis, while the inhibitor had the opposite effect. Importantly, the suppression of ferroptosis by miR-124-3p^in^-exos was significantly attenuated, confirming the critical role of miR-124-3p. We validated the predicted results in rat and human cell lines, respectively, and obtained consistent results, indicating that this modality of regulation is conserved. Notably, miR-124-3p^in^-exos still alleviated ferroptosis to some extent, suggesting that there might not be a single biomolecule acting in exosomes, thus the complete molecular composition and potential protective mechanisms of HM-exos will be explored in our future studies.

In vivo, treatment with HM-exos further inhibited the degree of ferroptosis in the liver after transplantation compared with that induced by M-exos, which also confirmed our bioinformatic prediction. In addition, HM-exos significantly increased the content of liver miR-124-3p and inhibited the elevated level of STEAP3, indicating that the inhibition of STEAP3 by delivery of miR-124-3p is an important mechanism by which HM-exos exert their protective role. Song et al. [46] found that HUCB-MSCs-exos inhibited the expression of *DMT1* by delivering miR-23a-3p, which suppressed cardiomyocyte ferroptosis after myocardial infarction; DMT1 is mainly responsible for transporting Fe^2+^ to the cytoplasm. We found that exosome treatment did not inhibit DMT1 expression, which might have been caused by excessive Fe^2+^ feedback leading to DMT1 upregulation. It should be noted that transformation of *Steap3* is not the only way to produce Fe^2+^ in cells. Nuclear receptor coactivator 4 (NCOA4) has also been proven to promote ferroptosis by degrading ferritin and releasing bound Fe^2+^ [47]. At the same time, our experiments showed that miRNAs are not the only substances in exosomes that are involved in this process, and other mechanisms might be involved in the regulation of cellular ferroptosis by exosomes.

The final outcome of ferroptosis is cell injury, which mediates the recruitment of neutrophils to damaged tissues in the early stage. Neutrophils are considered to be the central factor leading to post-reperfusion injury [34, 48]. Li et al. [49] proposed that ferroptosis in endothelial cells in the early stage after heart transplantation mediates the recruitment of neutrophils through the Toll like receptor 4 (TLR4)/ Toll like receptor adaptor molecule 1 (TICAM1, also known as TRIF)-dependent signaling pathway. Similarly, we found that neutrophil activation and chemokine signaling pathways were activated after transplantation, which led to a significant increase in infiltrating neutrophils in necrotic areas and a corresponding increase in the levels of inflammatory factors. However, HM-exo treatment reduced the infiltration of neutrophils and the level of inflammatory factors significantly, indicating that the inflammatory response is a downstream event of ferroptosis, i.e., ferroptosis mediates the infiltration of neutrophils, and the infiltrated neutrophils produce a large amount of ROS, which further amplifies the initial cell damage mediated by ferroptosis, leading to an aseptic inflammatory response.

Notably, the colloidal stability of exosomes during storage needs to be considered. A recent study showed that exosomes may undergo aggregation or degradation during prolonged freezing (− 80 ℃) or freeze-thawing, and when storage is strictly required, it is possible to suggest a short − 80 °C preservation time [50]. To avoid the impact of storage on the colloidal stability and biological properties of the exosomes, we chose to use freshly isolated or briefly frozen exosomes (less than 3 days) in the experiments. However, when large scale use or clinical trials are required, the impact of exosome isolation and storage on exosome stability needs to be addressed. Le et al. [51] demonstrated that the addition of alglucan enabled extracellular vesicles to maintain their colloidal stability well after cryopreservation at − 80 °C. In addition, a study suggested the use of lyophilization to maintain the stability of vesicles [52]. In future studies, we will focus on the possible effects of colloidal stability of exosomes and explore storage modes that meet the application criteria.

## Conclusion

In conclusion, our study showed that ferroptosis is involved in the IRI of LT with a severe steatotic donor liver, and that HO-1/BMMSC-derived exosomes could inhibit hepatocytes ferroptosis by delivering miR-124-3p to downregulate the level of *Steap3*, and finally reduce the IRI of the grafts. This provides new evidence for the clinical application of HO-1/BMMSC-derived exosomes in the field of organ transplantation, and provides new directions to solve the shortage of donor liver in the future.

## Supplementary Information

**Additional file 1: Figure S1.** Identification and biological properties of HO-1/BMMSCs. **Fig. S2.** HO-1/BMMSCs treatment attenuated IRI in steatotic grafts. **Fig. S3.** Steatotic IAR20 cells were more susceptible to ferroptosis after H/R treatment. **Fig. S4.** miR-124-3p inhibited ferroptosis in LO2 cells after H/R treatment. **Fig. S5.** Transfection efficiency of siRNA, plasmid and miR-124-3p-inhibitor. **Fig. S6.** DFO alleviates ferroptosis in IAR20 cells after H/R treatment. **Fig. S7.** HM-exo-mediated delivery of miR-124-3p attenuates ferroptosis in H/R-treated LO2 cells.

**Additional file 2: Table S1.** The sequences of miRNA mimic, miRNA inhibitor and small interfering RNA (siRNA). **Table S2.** Primer sequences for qRT-PCR. **Table S3.** PCR primer sequences for miRNA. **Table S4.** The GO analysis based on differentially expressed genes between PBS group and HM-exo group. **Table S5.** The KEGG analysis based on differentially expressed genes between PBS group and HM-exo group. **Table S6.** The KEGG analysis based on downstream genes of differentially expressed miRNAs in M-exo and HM-exo.

## Data Availability

All data and materials are available on request.

## Abbreviations

LT: Liver transplantation
IRI: Ischemia reperfusion injury
Fer-1: Ferrostatin-1
MSC: Mesenchymal stem cells
HO-1: Heme oxygenase oxygen-1
BMMSCs: Bone marrow mesenchymal stem cells
HO-1/BMMSC: Heme oxygenase oxygen-1 modified bone marrow mesenchymal stem cells
HM-exo: HO-1/BMMSC-derived exosomes
M-exo: BMMSC-derived exosomes
miRNAs: MicroRNAs
MCD: Methionine and choline deficient
TEM: Transmission electron microscopy
NTA: Nanoparticle tracking analysis
POD: Postoperative day
DEGs: Differentially expressed genes
GO: Gene ontology
KEGG: Kyoto Encyclopedia of Genes and Genomes
GSEA: Gene set enrichment analysis
H/R: Hypoxia and reoxygenation
MDA: Malondialdehyde
LIP: Labile iron pool
GSH: Glutathione
STEAP3: Prostate six transmembrane epithelial antigen 3
GPX4: Glutathione peroxidase 4
Ptgs2: Prostaglandin-endoperoxide synthase 2
4-HNE: 4-Hydroxynonenal
MPO: Myeloperoxidase
PBS: Phosphate-buffered saline
