# Supplementary material for: miR-124-3p delivered by exosomes from heme oxygenase-1 modified bone marrow mesenchymal stem cells inhibits ferroptosis to attenuate ischemia–reperfusion injury in steatotic grafts
Source: J Nanobiotechnology. 2022 Apr 22;20:196. doi: 10.1186/s12951-022-01407-8 (PMC9026664; doi:10.1186/s12951-022-01407-8)
Supplement: Supplementary file 1 — Additional file 1: Figure S1. Identification and biological properties of HO-1/BMMSCs. Fig. S2. HO-1/BMMSCs treatment attenuated IRI in steatotic grafts. Fig. S3. Steatotic IAR20 cells were more susceptible to ferroptosis after H/R treatment. Fig. S4. miR-124-3p inhibited ferroptosis in LO2 cells after H/R treatment. Fig. S5. Transfection efficiency of siRNA, plasmid and miR-124-3p-inhibitor. Fig. S6. DFO alleviates ferroptosis in IAR20 cells after H/R treatment. Fig. S7. HM-exo-mediated delivery of miR-124-3p attenuates ferroptosis in H/R-treated LO2 cells. [file 12951_2022_1407_MOESM1_ESM.docx]

**Supplementary figures and figure legends**


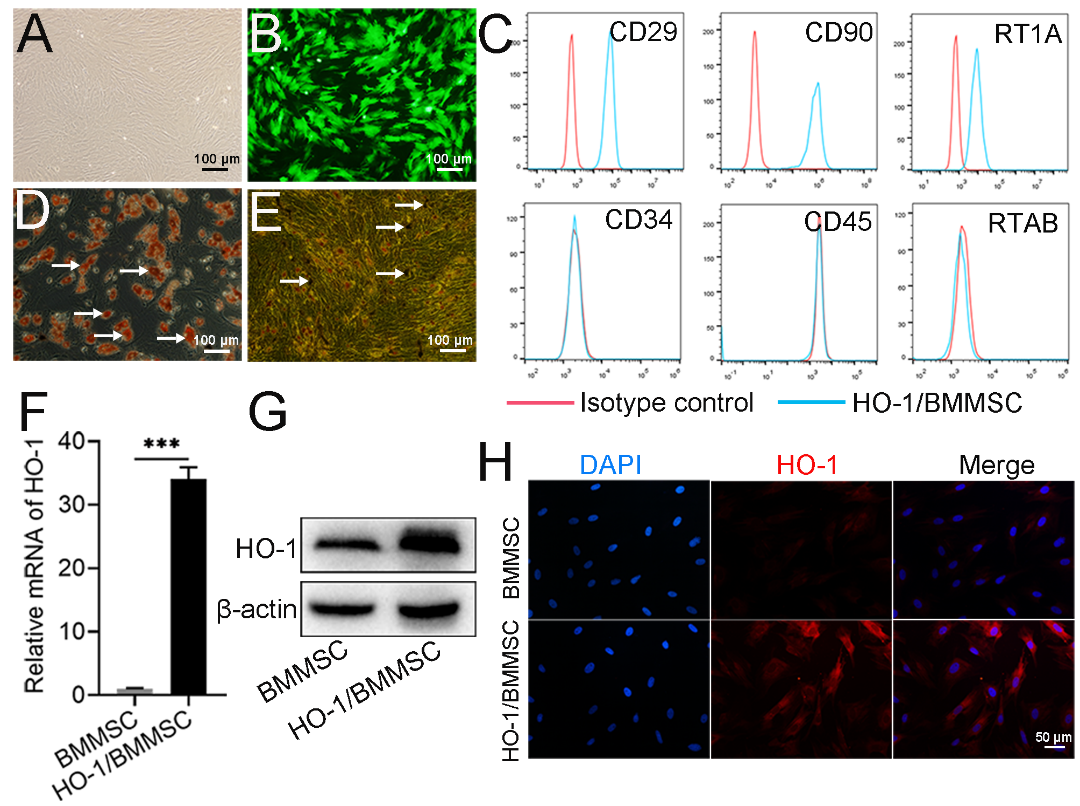


**Fig. S1. Identification and biological properties of HO-1/BMMSCs**

**(A)** BMMSCs transfected with HO-1 showed a long spindle shape. **(B)** BMMSCs transfected with HO-1 showed green fluorescence under a fluorescence microscope. **(C)** The specific markers of MSCs were detected by flow cytometry: CD29, CD90, and RT1A were positive; CD34, CD45, and RT1B were negative. **(D)** HO-1/BMMSCs induced adipogenic differentiation. Red lipid droplets (white arrow) were observed in the cells using Oil red O staining. **(E)** HO-1/BMMSCs induced osteogenic differentiation. Von Kossa staining showed black calcium deposition (white arrow). **(F-G)** HO-1 mRNA and protein levels in HO-1/BMMSCs were significantly higher than in BMMSCs. **(H)** Immunofluorescence confirmed that HO-1 protein levels in HO-1/BMMSCs were higher than in BMMSCs (n = 3). **P* < 0.05, ***P* < 0.01, ****P* < 0.001.


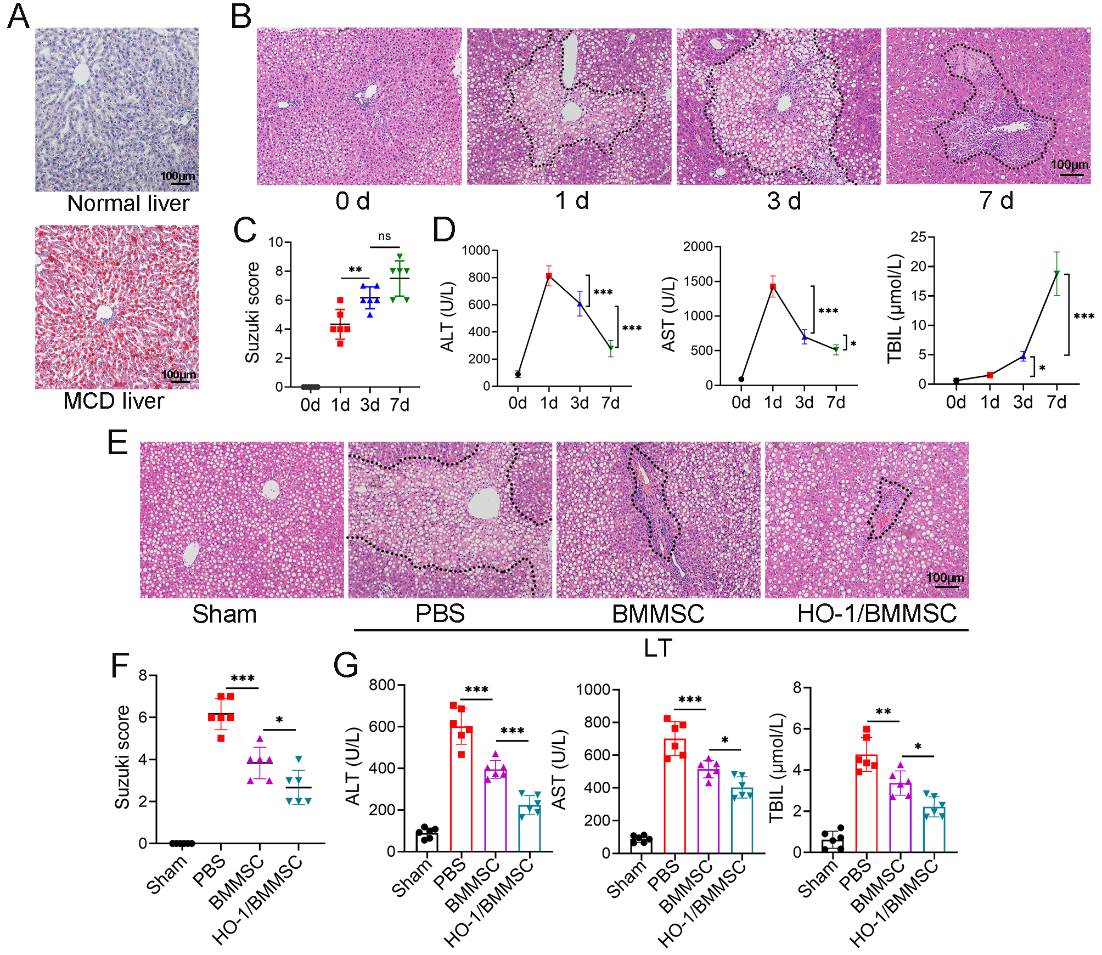


**Fig. S2. HO-1/BMMSCs treatment attenuated IRI in steatotic grafts**

**(A)** Oil red O staining of rat liver tissue from a normal liver and the MCD model. **(B)** Representative H&E staining images of liver tissues at POD1, POD3, and POD7, with the dashed black line representing necrotic areas. **(C)** Suzuki score of rat liver tissues at each time point. **(D)** The levels of serum ALT, AST, and TBIL in rats at each time point. **(E)** H&E staining of liver tissues on POD3 after treatment with PBS, BMMSCs, and HO-1/BMMSCs, the black dashed area represents the necrotic area. **(F)** Suzuki liver injury scores of rats in each group. **(G)** The levels of serum ALT, AST, and TBIL in each group (n = 6). **P* < 0.05, ***P* < 0.01, ****P* < 0.001. LT = Liver Transplantation; ns = not significant.


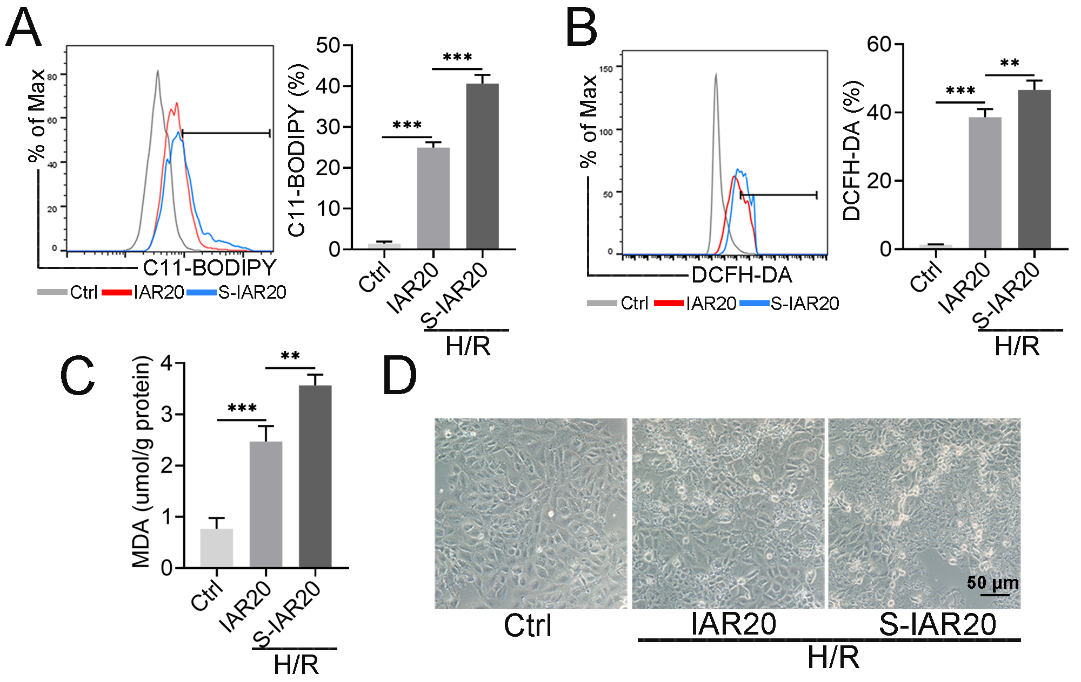


**Fig. S3. Steatotic IAR20 cells were more susceptible to ferroptosis after H/R treatment**

**(A)** The levels of Lipid-ROS (C11-BODIPY) in normal and steatotic IAR20 cells after H/R treatment. **(B)** The levels of ROS (DCFH-DA) in each group after H/R treatment. **(C)** The levels of MDA in each group after H/R treatment. **(D)** The morphology of cells was observed under a phase contrast microscope, and some cells were swollen and ruptured after H/R treatment (n = 3). **P* < 0.05, ***P* < 0.01, ****P* < 0.001.


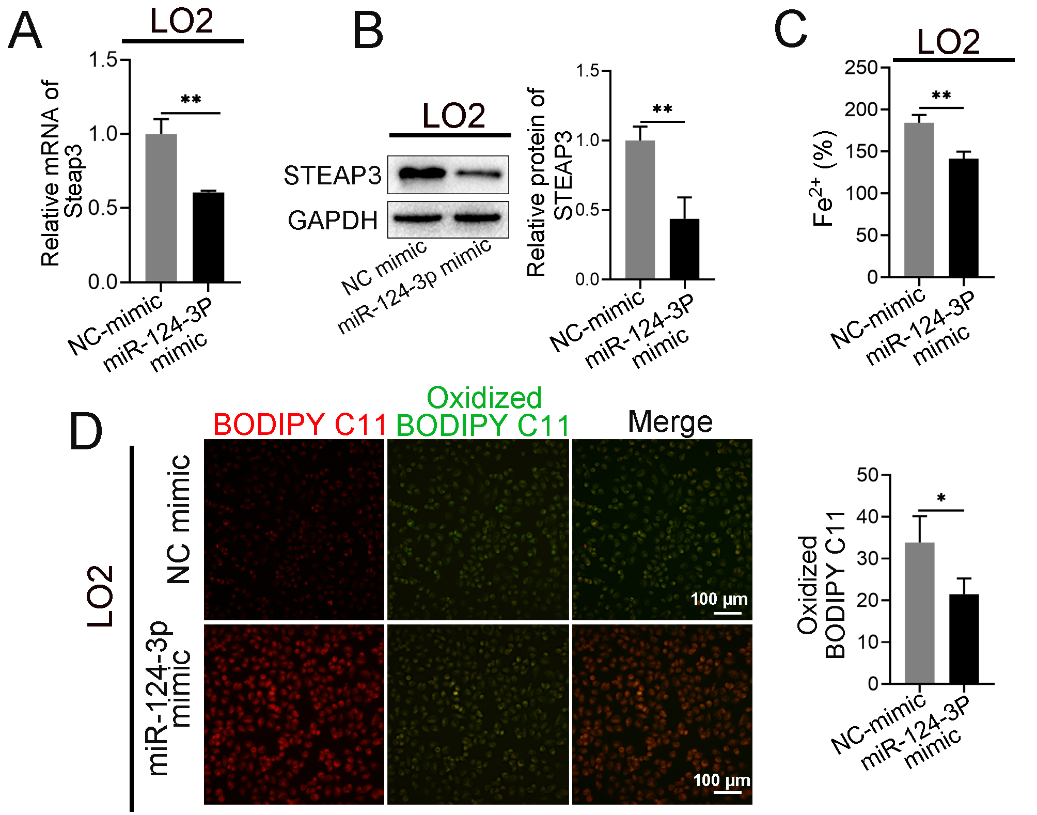


**Fig. S4. miR-124-3p inhibited ferroptosis in LO2 cells after H/R treatment**

**(A)** Relative expression levels of STEAP3 mRNA and protein **(B)** in LO2 cells overexpressing miR-124-3p. **(C)** LO2 cells were transfected with NC-mimic and mir‑124-3p-mimic respectively. The Fe^2+^ level of cells after H/R treatment was detected. **(D)** BODIPY581/591 staining showing the level of Lipid-ROS (Oxidized BODIPY-C11) in cells (n = 3). **P* < 0.05, ***P* < 0.01, ****P* < 0.001.


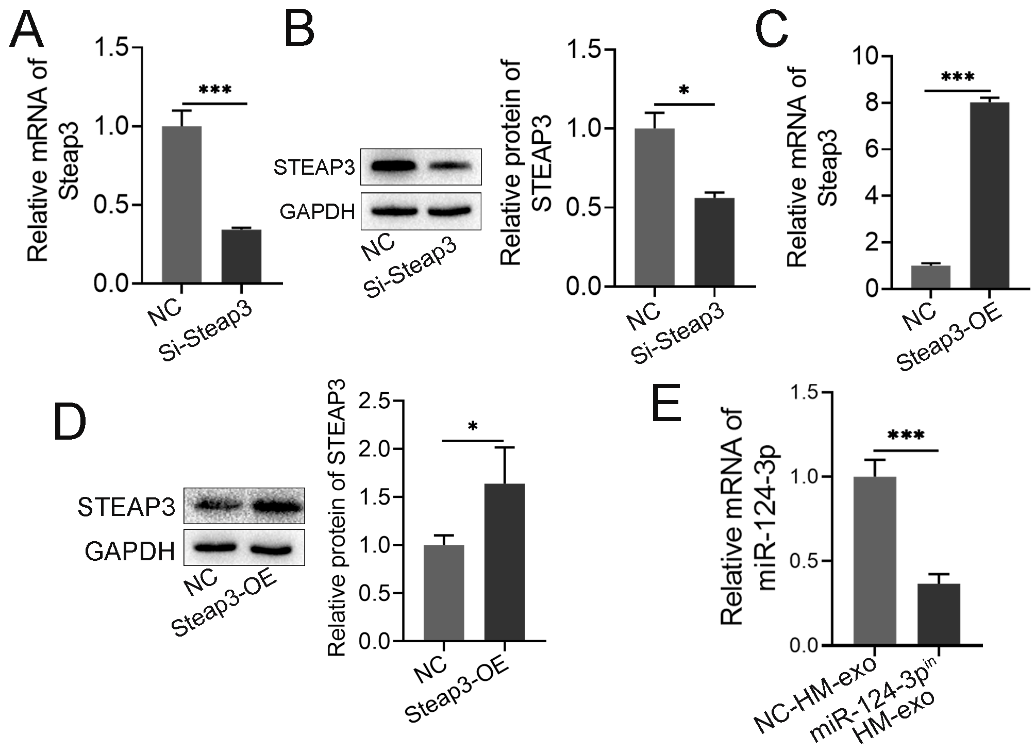


**Fig. S5. Transfection efficiency of siRNA, plasmid and miR-124-3p-inhibitor**

**(A)** Relative expression of STEAP3 mRNA and protein **(B)** in IAR20 cells transfected with *Steap3*-siRNA. **(C)** Relative expression of STEAP3 mRNA and protein **(D)** in IAR20 cells transfected with *Steap3*-OE. **(E)** HO-1/BMMSCs were transfected with NC inhibitor and miR-124-3p-inhibitor, respectively. The exosomes of the two groups were collected to detect the relative content of miR-124-3p in the exosomes (n = 3). **P* < 0.05, ***P* < 0.01, ****P* < 0.001. *Steap3*-siRNA = small interfering RNA targeting *Steap3*; *Steap*3-OE = Plasmid vector overexpressing *Steap3*; NC = negative control.


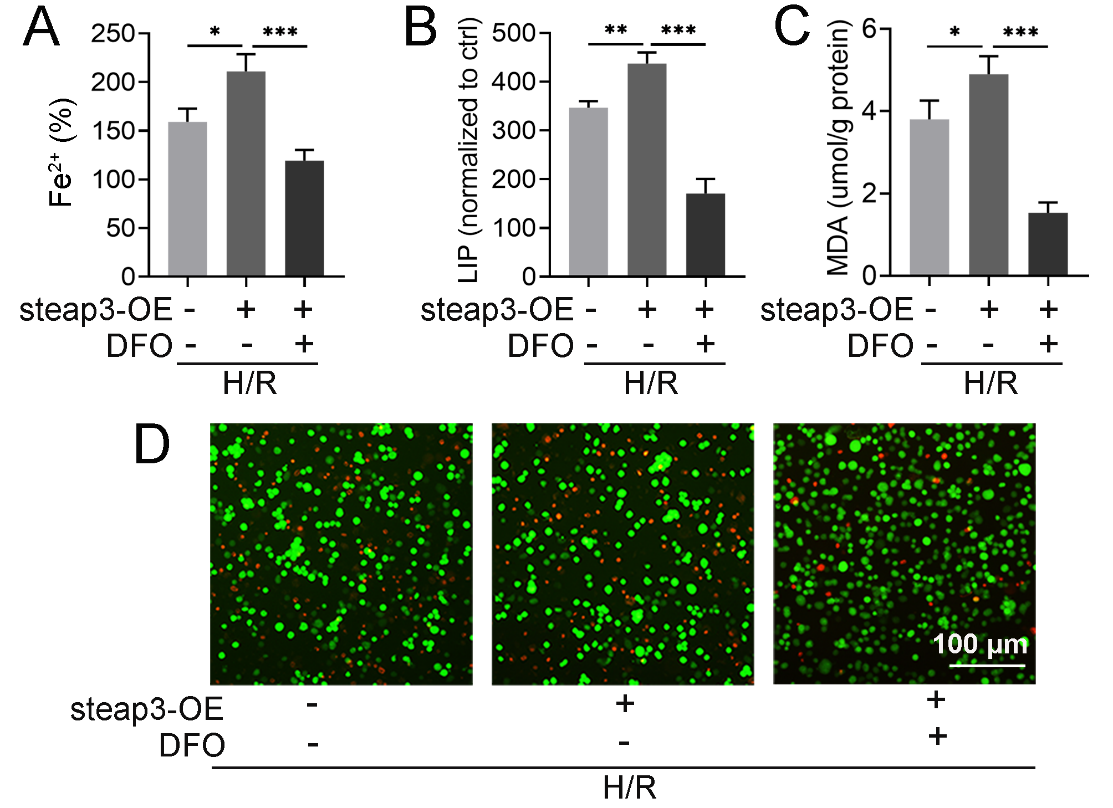


**Fig. S6. DFO alleviates ferroptosis in IAR20 cells after H/R treatment**

**(A)** IAR20 cells were transfected with *Steap3*-OE with or without DFO (100 μ M), the Fe^2+^ level of cells after H/R treatment was detected. **(B)** LIP and MDA **(C)** levels in each group. **(D)** PI/FDA staining identifying dead (red) and live (green) cells in each group. (n=3). **P* < 0.05, ***P* < 0.01, ****P* < 0.001 DFO: Deferoxamine mesylate; *Steap3*-OE = Plasmid vector overexpressing *Steap3*.


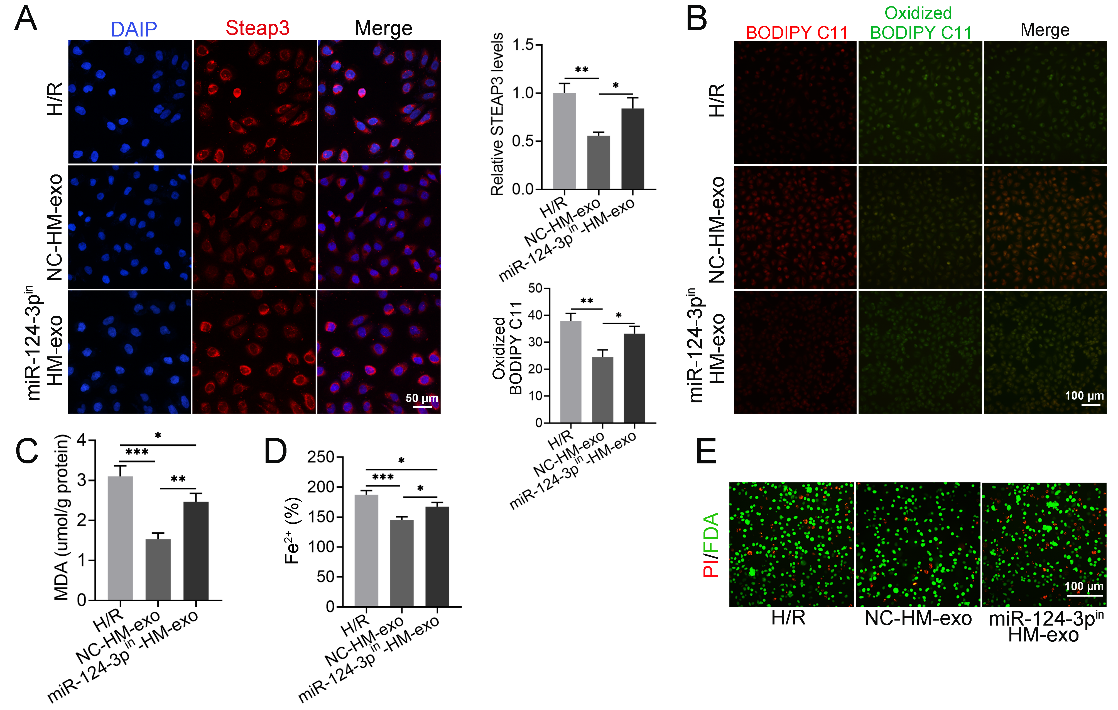


**Fig. S7. HM-exo-mediated delivery of miR-124-3p attenuates ferroptosis in H/R‑treated LO2 cells**

**(A)** NC-HM-exos and miR-124-3p^in^-HM-exos intervened in H/R treated LO2 cells respectively, and the levels of STEAP3 in the cells were detected by immunofluorescence. **(B)** BODIPY581/591 staining showing the level of Lipid-ROS (Oxidized BODIPY-C11) in each group. **(C)** MDA and Fe^2+^ **(D)** levels in each group. **(E)** PI/FDA staining identifying dead (red) and live (green) cells in each group (n = 3). **P* < 0.05, ***P* < 0.01, ****P* < 0.001.
