# Supplementary material for: miR-124-3p delivered by exosomes from heme oxygenase-1 modified bone marrow mesenchymal stem cells inhibits ferroptosis to attenuate ischemia–reperfusion injury in steatotic grafts
Source: J Nanobiotechnology. 2022 Apr 22;20:196. doi: 10.1186/s12951-022-01407-8 (PMC9026664; doi:10.1186/s12951-022-01407-8)
Supplement: Supplementary file 2 — Additional file 2: Table S1. The sequences of miRNA mimic, miRNA inhibitor and small interfering RNA (siRNA). Table S2. Primer sequences for qRT-PCR. Table S3. PCR primer sequences for miRNA. Table S4. The GO analysis based on differentially expressed genes between PBS group and HM-exo group. Table S5. The KEGG analysis based on differentially expressed genes between PBS group and HM-exo group. Table S6. The KEGG analysis based on downstream genes of differentially expressed miRNAs in M-exo and HM-exo. [file 12951_2022_1407_MOESM2_ESM.docx]

**Supplementary Tables**

**Table. S1.** The sequences of miRNA mimic, miRNA inhibitor and small interfering RNA (siRNA)

| **Gene** | **Sequences** |
| --- | --- |
| rno-miR-124-3p-mimic | sense: UAAGGCACGCGGUGAAUGCC  antisense: CAUUCACCGCGUGCCUUAUU |
| rno-NC-mimic | sense: UUCUCCGAACGUGUCACGUTT  antisense: ACGUGACACGUUCGGAGAATT |
| rno-miR-124-3p-inhibitor | 5′-GGCAUUCACCGCGUGCCUUA-3′ |
| rno-NC- inhibitor | 5′-CAGUACUUUUGUGUAGUACAA-3′ |
| hsa-miR-124-3p-mimic | sense: UAAGGCACGCGGUGAAUGCCAA  antisense: GGCAUUCACCGCGUGCCUUAUU |
| hsa-NC-mimic | sense: UUCUCCGAACGUGUCACGUTT  antisense: ACGUGACACGUUCGGAGAATT |
| hsa-miR-124-3p-inhibitor | 5′-UUGGCAUUCACCGCGUGCCUUA-3′ |
| hsa-NC-inhibitor | 5′-CAGUACUUUUGUGUAGUACAA-3′ |
| Rat-Steap3-siRNA | sense: GCUCUCCAGAGGUCAUCUUTT  antisense: AAGAUGACCUCUGGAGAGCTT |
| NC-siRNA | sense: UUCUCCGAACGUGUCACGUTT  antisense: ACGUGACACGUUCGGAGAATT |

**Table. S2.** Primer sequences for qRT-PCR

| **Target Gene** | **Primer sequence** |
| --- | --- |
| Rat-HO-1 | F: AGGAGATAGAGCGAAACAAGCAGAAC  R: GCTGTGTGGCTGGTGTGTAAGG |
| Rat-Steap3 | F: GTTGAGGAAGAAGTCTGGCGGATG  R: GCAATGGAAGGGATCGAGGTAACC |
| Rat-ptgs2 | F: TGTCAAAACCGAGGTGTATGTA  R: AACGTTCCAAAATCCCTTGAAG |
| Rat-β-actin | F: CGCGAGTACAACCTTCTTGC  R: ATACCCACCATCACACCCTG |
| Has-Steap3 | F: CTACTCTTCACTGTGCAGTCTC  R: GAGATGACATTGAAGGCCTTGA |
| Has-β-actin | F: CACCCAGCACAATGAAGATCAAGAT  R: CCAGTTTTTAAATCCTGAGTCAAGC |

**Table. S3.** PCR primer sequences for miRNA.

| **Gene (miRNA)** | **RT-primer** | **Sense primer** | **Antisense primer** |
| --- | --- | --- | --- |
| rno-miR-124-3p | GTCGTATCCAGTGCAGGGTCCGAGGTGCACTGGATACGACGGCATTC | TGCGGTAAGGCACGCGGTGAATG | CCAGTGCAGGGTCCGAGGT |
| has- miR-124-3p | GTCGTATCCAGTGCAGGGTCCGAGGTGCACTGGATACGACGGCATTC | TGCGGTAAGGCACGCGGTG | TGCAATGATGAAAGGGCAT |
| U6 | TCACGAATTTGCGTGT | CGCTTCGGCAGCACAT | ATTTGCGTGTCATCCTTGC |

**Table. S4.** The GO analysis based on differentially expressed genes between PBS group and HM-exo group.

| **Term ID** | **Term description** | **Gene Number** | **Rich Ratio** | **Q-value** |
| --- | --- | --- | --- | --- |
| GO:0030593 | neutrophil chemotaxis | 35 | 0.49 | 7.33E-08 |
| GO:0055114 | oxidation-reduction process | 182 | 0.25 | 1.02E-07 |
| GO:0006869 | lipid transport | 33 | 0.36 | 3.79E-04 |
| GO:0010942 | positive regulation of cell death | 27 | 0.39 | 4.20E-04 |
| GO:0006749 | glutathione metabolic process | 23 | 0.38 | 0.00208294 |
| GO:0032930 | positive regulation of superoxide anion generation | 13 | 0.50 | 0.003881988 |
| GO:0042119 | neutrophil activation | 8 | 0.57 | 0.0194409 |
| GO:0055072 | iron ion homeostasis | 14 | 0.40 | 0.02139253 |
| GO:0045730 | respiratory burst | 6 | 0.60 | 0.04771946 |
| GO:0046321 | positive regulation of fatty acid oxidation | 6 | 0.60 | 0.04771946 |

**Table. S5.** The KEGG analysis based on differentially expressed genes between PBS group and HM-exo group.

| **Term ID** | **Pathway Term description** | **Gene Number** | **Rich Ratio** | **Q-value** |
| --- | --- | --- | --- | --- |
| 4060 | Cytokine-cytokine receptor interaction | 91 | 0.34 | 5.88E-09 |
| 4621 | NOD-like receptor signaling pathway | 67 | 0.37 | 2.58E-08 |
| 4062 | Chemokine signaling pathway | 63 | 0.35 | 4.41E-07 |
| 4670 | Leukocyte transendothelial migration | 40 | 0.35 | 1.10E-04 |
| 590 | Arachidonic acid metabolism | 30 | 0.37 | 3.81E-04 |
| 4660 | T cell receptor signaling pathway | 35 | 0.32 | 0.001420201 |
| 480 | Glutathione metabolism | 25 | 0.35 | 0.002654093 |
| 4611 | Platelet activation | 35 | 0.27 | 0.02983239 |
| 4216 | Ferroptosis | 14 | 0.34 | 0.03984999 |
| 4066 | HIF-1 signaling pathway | 31 | 0.27 | 0.04781911 |

**Table. S6.** The KEGG analysis based on downstream genes of differentially expressed miRNAs in M-exo and HM-exo

| **Term ID** | **Pathway Term description** | **Gene Number** | **Enrichment score** | **p-value** |
| --- | --- | --- | --- | --- |
| 05215 | Prostate cancer | 7 | 5.23 | 0.000360746 |
| 04130 | SNARE interactions in vesicular transport | 4 | 8.70 | 0.001068319 |
| 04622 | RIG-I-like receptor signaling pathway | 5 | 5.98 | 0.001421276 |
| 04068 | FoxO signaling pathway | 7 | 3.86 | 0.002173095 |
| 05202 | Transcriptional misregulation in cancers | 8 | 3.42 | 0.002304529 |
| 04668 | TNF signaling pathway | 6 | 4.06 | 0.003516511 |
| 01521 | EGFR tyrosine kinase inhibitor resistance | 5 | 4.54 | 0.004761798 |
| 04350 | TGF-beta signaling pathway | 5 | 4.27 | 0.006175808 |
| 04151 | PI3K-Akt signaling pathway | 11 | 2.36 | 0.006762957 |
| 04657 | IL-17 signaling pathway | 5 | 3.94 | 0.00861888 |
| 04915 | Estrogen signaling pathway | 6 | 3.31 | 0.009408705 |
| 04115 | p53 signaling pathway | 4 | 4.35 | 0.013257178 |
| 05206 | MicroRNAs in cancer | 6 | 3.05 | 0.01368297 |
| 04216 | Ferroptosis | 3 | 5.52 | 0.01671244 |
| 04150 | mTOR signaling pathway | 6 | 2.87 | 0.018070663 |
